# The Role of Medicinal Cannabis in Clinical Therapy: Pharmacists' Perspectives

**DOI:** 10.1371/journal.pone.0155113

**Published:** 2016-05-12

**Authors:** Sami Isaac, Bandana Saini, Betty B. Chaar

**Affiliations:** Faculty of Pharmacy, The University of Sydney, Sydney, New South Wales, Australia; Penn State College of Medicine, UNITED STATES

## Abstract

**Background:**

Medicinal cannabis has recently attracted much media attention in Australia and across the world. With the exception of a few countries, cannabinoids remain illegal–known for their adverse effects rather than their medicinal application and therapeutic benefit. However, there is mounting evidence demonstrating the therapeutic benefits of cannabis in alleviating neuropathic pain, improving multiple sclerosis spasticity, reducing chemotherapy induced nausea and vomiting, and many other chronic conditions. Many are calling for the legalisation of medicinal cannabis including consumers, physicians and politicians. Pharmacists are the gatekeepers of medicines and future administrators/dispensers of cannabis to the public, however very little has been heard about pharmacists’ perspectives. Therefore the aim of this study was to explore pharmacists’ views about medicinal cannabis; its legalisation and supply in pharmacy.

**Methods:**

Semi-structured interviews with 34 registered pharmacists in Australia were conducted. All interviews were audio-recorded, transcribed *ad verbatim* and thematically analysed using the NVivo software.

**Results:**

Emergent themes included stigma, legislation, safety and collaboration. Overall the majority of pharmacists felt national legalisation of a standardised form of cannabis would be suitable, and indicated various factors and strategies to manage its supply. The majority of participants felt that the most suitable setting would be via a community pharmacy setting due to the importance of accessibility for patients.

**Discussion:**

This study explored views of practicing pharmacists, revealing a number of previously undocumented views and barriers about medicinal cannabis from a supply perspective. There were several ethical and professional issues raised for consideration. These findings highlight the important role that pharmacists hold in the supply of medicinal cannabis. Additionally, this study identified important factors, which will help shape future policies for the successful implementation of medicinal cannabis in healthcare. We recommend that these views and strategies be incorporated in the development of policies and legislations.

## Introduction

For over 4000 years cannabis more commonly known as marijuana, has been used medicinally, recreationally and in religious ceremonies in cultures across the globe [1]. Well known in the public arena, recreational cannabis is the leisurely use of cannabis for its euphoriant effect. Medicinal cannabis, on the other hand is regarded as the use of cannabis ‘to achieve a curative or remedial effect’ on the symptoms of a medical condition [2]. From a healthcare perspective, medicinal use of cannabis would refer to use based on a prescription or recommendation by a registered physician, for a known medical condition, that has evidence demonstrating its indication and efficacy [3].

For many years, cannabis has been viewed as an illicit substance and this negative repute has hindered efforts to conduct research into its therapeutic benefits. A plethora of literature exists investigating the abuse, misuse and side effect profile of cannabis, in the realms of addiction, mental cognition and schizophrenia.

More recently however, there has been a gradual increase of research into the therapeutic benefits associated with the medicinal use of cannabis. With a greater number of well designed trials providing evidence to support its use for spasticity, chronic neuropathic pain and chemotherapy-induced nausea and vomiting, cachexia, as well as appetite stimulation in HIV/AIDS infection [4]. The expanding body of research into the medicinal application of cannabis has initiated the development of marketable forms of cannabis internationally, as well as rapid policy making by governing bodies worldwide.

A systematic and meta-analysis into the therapeutic applications of cannabis provided moderate-quality evidence to support the use of cannabinoids for the treatment of chronic pain and spasticity. For conditions other than pain and spasticity (such as nausea and vomiting, weight loss in HIV infection, sleep disorders and Tourette syndrome), minor improvements were noted [4] and more evidence is emerging as more stringent investigations are being undertaken.

Globally, the type and intent of legislation governing cannabis use is complex and varied with focus on both general and medicinal use. In the case of medicinal cannabis, countries including the UK, Denmark, Czech Republic, Austria, Sweden, Germany, and Spain [3] have all formally approved use of cannabis based products of one form or another (Table 1), **[5]** thus decriminalising its therapeutic use.

**Table 1:** Forms of Cannabinoids, their effects and any registered products (5).

Cannabinoids	Source	Pharmacological Effects	Registered Products
Anandamide (Δ8-THC)	Endogenous ligand	Memory/Cognition, Motivation and Pleasure	N/A
Δ9-tetrahydrocanabinol (THC)	Natural/ Botanical	Psychoactive, ↓ Memory/Cognition, ↑ Pleasure and Appetite stimulation	See Nabiximols
Cannabidiol (CBD)	Natural/ Botanical	Non-psychoactive, Antiemetic and Antispasmodic	See Nabiximols
Cannabinol (CBN)	Natural/ Botanical	Non-Psychoactive, Anti-inflammatory and Immunosuppressive	N/A
Nabiximols	Combination of THC and CBD	See THC & CBD	Sativex = 2.7mg THC + 2.5mg CBD (oromucosal spray)
Dronabinol	Synthetic THC	Appetite stimulating	Marinol = 2.5mg, 5mg, 10mg (tablet/capsule)
Nabilone	Synthetic analogue of THC	Antiemetic and Antispasmodic	Cesamet = 1mg (capsule)

In the United State (US) to date, 18 states and Washington DC have legalised recreational cannabis use, and 23 states in total have legalised the medicinal use of cannabis [6]. The US Foods and Drugs Administration (FDA) has yet to approve the marketing of products containing or derived from botanical marijuana extract–despite its legislative status. There are, however, approved synthetic cannabis derivatives formulated such as *dronabino*l, the marketed delta-9-tetrahydrocannabinol (THC) analogue registered as Marinol, for the therapeutic treatment of anorexia in AIDS patients. Also, Cesamet containing the synthetic THC analogue *nabilone*, which is approved as a last line antiemetic. [7] The European Medicine Agency has granted registration of dronabinol [8] for the treatment of central and peripheral neuropathic pain. It has also registered the *nabiximol (*Sativex) [9], a combination of cannabidiol and THC, for the indicated treatment of spasticity in multiple sclerosis and *nabilone* [10] for the treatment of amyotrophic lateral sclerosis.

In the Australasian sector, the Australian register of Therapeutic Goods (TGA) has registered Sativex for future use, but the distributor Novartis™ has made the commercial decision not to make it available [6]. However as of 24^th^ February 2016, The Australian parliament passed new national laws and amendments to the Narcotics Drug Act 1967 to allow for the controlled cultivation of cannabis for medical and scientific purposes in Australia [11]. This followed the Victorian state government’s introduction of legislation into parliament to legalise medicinal cannabis based on the advice of the Victorian Law Reform Commission’s Report on Medicinal Cannabis [12]. These legislations are designed to allow access for those in need while limiting abuse and diversion potential. In New Zealand, medicinal cannabis remains illegal, though the recent approval for the use of Elixinol (a cannabidiol usually marked as a dietary supplement based on anti-oxidant properties of CBD) in a coma patient suffering status epilepticus actioned by the health minister on “compassionate grounds” has sparked a spate of discussion in the medical community. [13–15]

Worldwide, the growing development of cannabis-based medicines has led to greater discussion among patients, prescribers and policy makers. As prescribers of cannabis, physicians’ perspectives have been documented in studies conducted in the US [16], Canada [17] and Israel [18]. These results illustrate how medical practitioners’ views have been explored in different national settings especially in Organisation for Economic Cooperation and Development (OECD) countries. However, in such countries ethical principles in medicine mandate a degree of separation between prescribing a drug and its supply; thus necessitating the need for independent channels of distribution. In most instances this is the role of the pharmacy profession. Thus–should medicinal cannabis be legalised–pharmacists would be responsible for the stocking, handling, ethical supply, counselling and overseeing the safe use of medicinal cannabis. This makes their professional support, opinion and perspective a fundamental aspect to be explored in order to ensure medicinal cannabis is implemented successfully.

The pharmacy profession itself has championed many public health services across several OECD nations in order to better the community. A key example in the UK and Australia is the implementation of fundamental harm-minimisation programmes such as the needle exchange program, which aims to reduce the transmission of blood borne viruses among intravenous drug users [19]. The opioid substitution therapy (OST) is another program aimed at reducing risk behaviours and illicit drug use in individuals [20]. The provision of flu vaccinations by pharmacists across Europe, Canada, the UK and Australia is an expanded public health prevention service and helps to reduce the burden of disease costs on healthcare systems. In addition to these services pharmacists have been central in the supply of the emergency contraception, aiming to reduce the risk of unwanted pregnancies, especially in young adults [21].

In the implementation and supply of each of these public health measures pharmacists’ perspectives and pragmatic recommendations have been important. Given the ongoing debate and controversy surrounding medicinal cannabis, the viewpoint of the pharmacy profession is just as important for the successful implementation and delivery of medicinal cannabis to patients worldwide. This is the first study to investigate pharmacists’ perspectives on the supply and legalisation of medicinal cannabis.

## Aim

To explore and investigate Australian pharmacists’ views on medicinal cannabis and their role in its supply.

## Objective

To explore pharmacists’ perspectives on the facilitators and barriers with respect to:

- The legalisation of medicinal cannabis in Australia
- Dispensing medicinal cannabis in community pharmacies
- The support needs in relation to provision of medicinal cannabis in community pharmacies

## Methods

### Ethics Statement

Prior to commencement of the study (July–November 2015), approval was obtained from the University of Sydney Human Research Ethics Committee (Ref No. 2015/591). This included approval for the method of obtaining participant’s consent, utilizing an approved Participant Information Statement [PIS].

All participants were required to sign a formal standardized Consent Form before starting the interview and audio-recording, as per University of Sydney HREC requirements. Signed Consent forms, constituting evidence of informed consent, from every participant were collected and retained. These were stored in hard copies in a dedicated locked cabinet in the supervising researcher's office.

### Recruitment

The sampling strategy involved a convenience sampling of Australian pharmacists. To enable the sampling of a wide variety of views, the inclusion criteria were that the interviewees were currently registered with the Australian Health Practitioner Regulation Agency as pharmacists, and willing to express their views on the legalisation of medicinal cannabis. It also employed a purposive sampling of a subset of Leading Representatives of Professional Organisations (LRPO) and was followed by passive snowballing as a result of individual requests to participate. LRPO’s were identified based on their credentials, the prominence of their role in the Australian pharmacy profession, and their ability to influence the industry as professional leaders and representatives. Their contact details were found in the public domain from various Internet websites and professional journals. Recruitment was initiated via email, phone and face-to-face invitation circulation, along with advertisements placed on professional society newsletters and social media sites of professional organisations. A broad range of locations were targeted in order to capture a variety of perspectives based on location, practice environment and experience. We sought the perspective of community pharmacists and key stakeholders of the profession.

### Design of interview

An interview protocol (Table 2) was developed based on research literature on medicinal cannabis (4, 16–18) and practice experience of the researching team. The semi-structured interviews incorporated open-ended questions to enable the exploration of new ideas with prompts to allow deeper probing and expansion of key issues relating to medicinal cannabis. For participants who required greater knowledge or awareness about medicinal cannabis, clinical research papers including a meta-analysis was provided to them for greater familiarity with the topic, before re-commencing the interview at a later scheduled time.

**Table 2:** Interview Protocol.

QUESTIONS	PROMPTS
What are your general thoughts about medicinally used cannabis?	Understanding of legal status?
	Various uses?
	Evidence about its potential for medicinal use?
Do you believe it might be beneficial to make medicinal cannabis legal or should it remain illicit?	Effect on pharmacy practice?
	Would you be happy to dispense medicinal cannabis if made available?
	Potential problems with medicinal cannabis from a pharmacist’s point of view?
Do you think the substance “cannabis” attracts a stigma?	Links with its side effects when used illicitly?
	Based on evidence?
	Is medicinal cannabis in capsule form any different from other medicines?
What do you believe pharmacists should be saying in this debate?	Role of Pharmacy profession?
	Other healthcare professionals’ roles?

**Fig 1:**
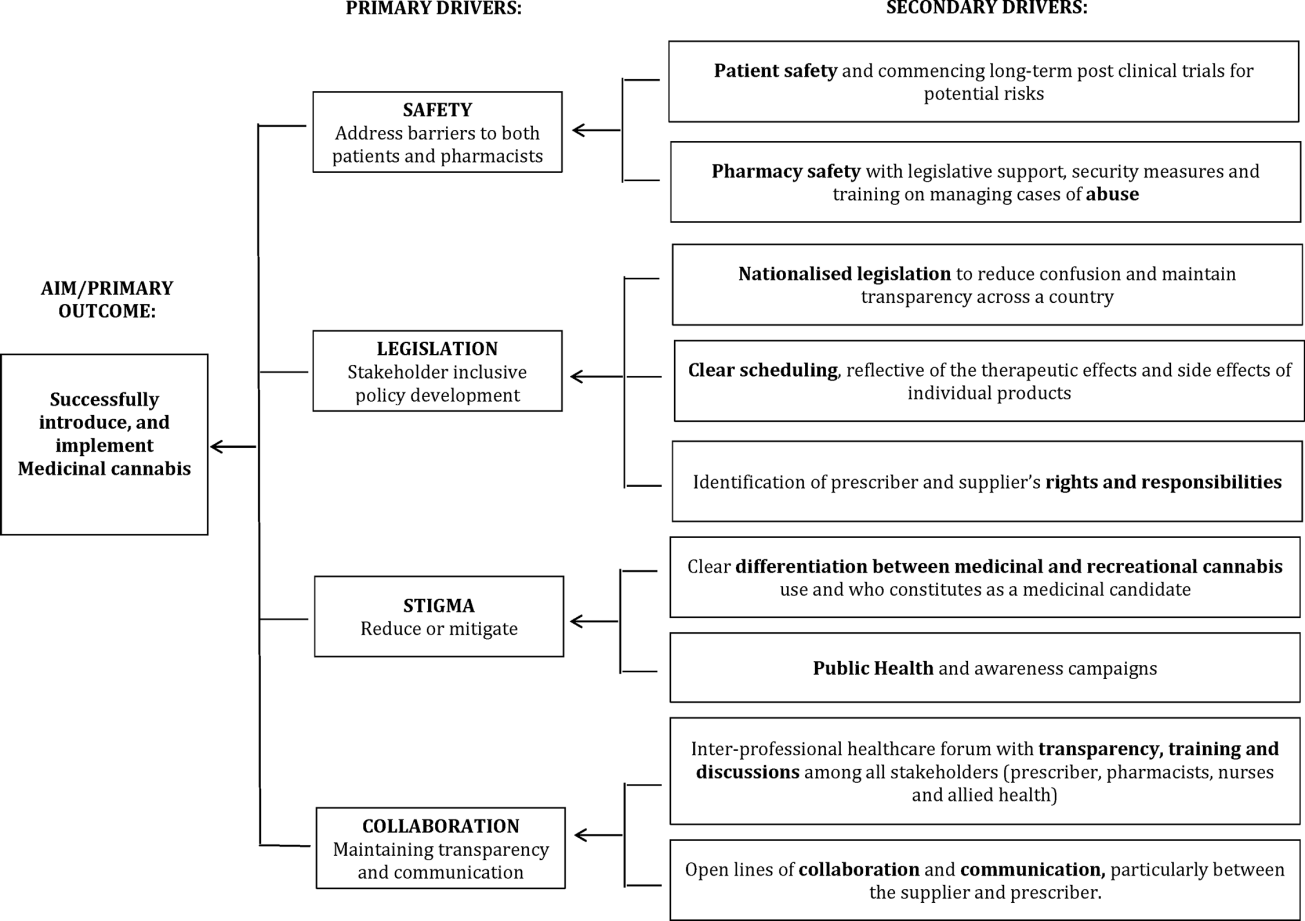
Driver diagram of emergent themes from interviews (n = 34)

For uniformity, the interviews were conducted by one interviewer (SI) and were between 10–30 minutes in length each. They were audio-recorded following participant consent, transcribed *ad verbatim* and de-identified. Data were then entered into NVivo (QSR Version 10.0.3 Mac) software for coding. For quality control and to ensure reasonably objective analyses, the research team independently read and coded transcripts into themes to create a coding scheme for thematic analysis [22]. Constant comparison of interviews helped extract key perspectives and ensured a level of consistency and reliability of analysis. These key themes were then used to generate a driver diagram as seen in **Fig 1.**

## Results

### Participant demographics

A total of 34 respondents met inclusion criteria and participated in the study. The gender distribution was equivalent, and their level of practice experience varied (Table 3). Registered pharmacists from across Australia were interviewed, but a greater proportion were pharmacists practicing in the state of New South Wales. In regards to primary roles between practicing pharmacists, academics and LRPOs, the majority of participants were solely practicing pharmacists. Although concordant with other representative data, the views, strength of conviction and proactive ideas of LRPO participants emanated more resoundingly when compared to other participants. This was determined via linguistic analysis of the recorded interviews and interpretation of verbal cues.

**Table 3:** Demographic of registered pharmacists’ interviewed (n = 34).

Characteristics		n	%
**Gender**	Male	17	50
	Female	17	50
**Age**	20 to 29	17	50
	30 to 39	8	23
	40 to 49	4	12
	50 to 59	3	9
	60+	2	6
**Number of years in practice**	≤ 1	4	12
	1 to 5	11	32
	6 to 10	6	18
	11 to 15	4	12
	16 to 20	1	3
	≥ 21	8	23
**Practice Location**	New South Wales	27	79
	Victoria	5	15
	South Australia	1	3
	Western Australia	1	3
**Primary Roles**	Practicing Pharmacists	25	73
	Academia	3	9
	Leading representatives of professional organisations (LRPO)	6	18

Interestingly, there were varying levels of knowledge across the differing demographics. Different patterns of awareness were observed, as pharmacists in academia had greater pharmacological and pharmacotherapeutic knowledge while LRPOs appeared to have a greater sense of clinical and regulatory awareness than those in other roles.

It was also acknowledged by LRPO participants that professional organisations in pharmacy–like theirs–should take responsibility for the ongoing training and support of members of the profession when it comes to the legalisation of medicinal cannabis.

Overall, the majority of participants expressed support and encouragement for the legalisation of medicinal cannabis with a sense of duty of care to their patients.

“It’s quite exciting. There is finally going to be a treatment option for those that up until now had no hope and no treatment. And as a pharmacist… patients’ health and well-being is the upmost of our priority … giving them a better quality of life is something that basically we work for every day.” (Interview #3)

The results are presented under the five following emergent themes: *role of the pharmacist*, *legislation*, *safety*, *stigma and collaboration* (Table 4). Each theme was deduced from the data of quotes extracted from the interview transcripts.

**Table 4:** Themes and sub-themes describing pharmacists’ views on the legalisation and implementation of medicinal cannabis.

Themes	Sub-themes
Role of Pharmacists	Significance
Legislation	Scheduling
	Nationalisation
	Quality Assurance
	Access
Safety	Patient safety
	Risk of Abuse (SE)
	Safety of the Pharmacy
Stigma	Public
	Media
Collaboration	Professional Training & Public Awareness
	Unified Communication

### Role of the Pharmacist

The majority of participants expressed agreement that pharmacists would play an essential role in providing legitimate access to medicinal cannabis. As drug specialists, participating pharmacists identified their role as central to the drugs supply, use and safekeeping.

“We need to have our input into the matter, I think that it is very important. You know we are the ones to most likely dispense and supply it.“(Interview #18)

They also acknowledged, successful implementation of medicinal cannabis programs require input from the profession in this contemporary debate and discussions amongst all involved.

“We are all part of the healthcare professional team and in order for us to help the patient we need to actually work hand-in-hand together and have all different types of opinions amalgamated into one.” (Interview #22)

### Legislation

#### Scheduling

The majority of the participants expressed the view that medicinal cannabis would be best introduced as a controlled substance, which under Australian regulation is categorised as a dangerous drug or schedule 8 (S8) as opposed to prescription only medicines or schedule 4 (S4) drugs [23].

Participants felt that this controlled scheduling would be best suited for medicinal cannabis due to its perceived propensity to be misused. Also based on past experiences with the OST and misuse of other medicines such as pseudoephedrine for illicit purposes, pharmacists expressed a desire for stringent guidelines as a means of legislative support.

“Initially it would be like a S8 and I think that's the appropriate place for it. S8 gives the pharmacist some comfort about the level of legislation behind it; checking the medication and making sure doctors write the prescriptions properly.” (Interview #6)

On the other hand some participants who had a greater knowledge on the cannabinoid constituents and their pharmacological effects conveyed a lesser focus on controlled scheduling, rather a greater focus on its accessibility as a prescription drug.

“I don’t see it as a S8 medicine, based on its use. I don’t really see it as the same thing as methadone. I see it more as a schedule 4.” (Interview #17)

In addition to the legislation, some participants suggested the development of comprehensive recording systems (e.g. where medication supply is recorded in pharmacy registers) to be incorporated. Parallels were drawn with current systems for recording of supplied medications including: staged supply, methadone subsidiaries, and the clozapine portal. These pharmacists felt that in order to fulfil their duty of care to “do no harm” [24] as healthcare professionals a stringent protocol with recording systems in place would be needed.

#### Nationalisation

Some participants stated that the success of implementation of legal medicinal cannabis supply would depend on a nationalised framework. Pharmacists’ support for a nationalised framework was to ensure a level of consistency, uniformity and standardisation across the country (i.e. to avoid inter-state variations).

“Establishing a nationalised system and accompanying that with the current E-Health scripts … that would help manage this well.” (Interview #8)

#### Quality Assurance

Participants with a broad knowledge of all the multiple constituents of cannabis stressed a need to have stringent quality assurance protocols. They expressed the need for a standard homogenous stable formulation of a high pharmaceutical grade, regardless of manufacturer.

“A committee should recommend a standard formulation containing specific moieties (sic.) that can be prepared and then that’s the one standard formulation that’s made available nationally.” (Interview #1)

Conversely, a portion of those interviewed revealed a confidence in existing regulatory bodies to govern the standardisation of medicinal cannabis and its key components.

“As long as the active ingredient are produced or packaged by a TGA licensed facility, then I don’t see why there’d be a problem.” (Interview #11)

Majority of participants (even those who worked in pharmacies that identified as compounding pharmacies) felt strongly about the initiation of medicinal cannabis as a standardised pharmaceutical product in order to preserve the medications quality control and minimise any risk of harm to the patient associated with compounded forms of cannabis.

“You can’t just grow a plant because there are variations in the plant itself and in the growing conditions. I see it as being a medicinally used product… I guess much along the lines that digitalis was standardised and used. I mean over the years, pharmacology has developed from plants and it’s a lot better when we have standardised and know what the ingredients are.” (Interview #31)

#### Access

Several avenues for access to medicinal cannabis were proposed. The majority of participants felt that the most suitable setting would be via a community pharmacy setting due to the importance of accessibility for chronic and palliative patients.

“It should be within a community setting. I think that all palliative care should be… in terms of accessibility, within the community is best.” (Interview #6)

This was followed by the suggestion of staged implementation, with supply initiating at clinics or hospitals before being introduced to a community setting.

“Initially in a clinic setting and then following good feedback and positive outcomes in a community setting… because it is more readily available.” (Interview #7)

Some participants preferred cannabis to be supplied in a hospital environment with the key reason cited being a more specialised team monitoring its use. A few participants making this suggestion, also proposed a clinic setting like that used for methadone initiation would minimise the potential for cannabis abuse.

A number of participants were indifferent to the location of supply, suggesting that it could be successfully supplied in a multiple number of settings in order to make it accessible to all patients in various locations and with various needs. A few participants suggested a specialised cannabis supplier model similar to those existing overseas as means of cannabis supply.

### Safety

#### Patient Safety (SE

A number of participants were concerned about potential long-term effects of medicinal cannabis with risks associated with cognitive impairment and psychosis. However, most participants mentioned that all medicines have risks involved and it is a matter of weighing up those risks with the benefits for each individual undergoing treatment.

“There are potential harms associated with its long-term use, like with any medicine. There is no doubt about that. But throwing the blanket over the whole thing and saying no we can’t use it because of that, is kind of a way out and unethical.” (Interview #29)

#### Risk of Abuse

A few participants perceived the gaps in current regulations and recording systems as an opportunity for abuse and misuse of controlled substances. They emphasised the need to either have more stringent safe keeping protocols or even have special formulations to avoid these perceived risks.

“If it becomes legalised than it may be easier than before to be abused.” (Interview #20)

“I’m thinking direct contact with the doctor every time something has been prescribed, no repeats at all, just constant follow ups with each healthcare professional to make sure it’s not being abused.” (Interview #27)

On the other hand, the majority of participants didn’t deem this perceived risk as a barrier to the introduction of medicinal cannabis. The mainstream outlook on medicinal cannabis is that such a formulation for medicinal use makes the risk of abuse or diversion potential almost insignificant.

“From my understanding these medicinal products are not that divertible or desirable…due to their formulation, so the hype of not using it (in fear of abuse) is unjustified” (Interview #30)

Many held the firm view that the profession was one of primary care and prevention and was well equipped to manage and assist those who need it most.

“We are healthcare professionals and if we identify abuse then we refer people to the correct avenues. That’s our role as pharmacists. We are not here to make people who have a problem with medicines feel like they’re criminals and we refer them to appropriate health facilities.” (Interview #11)

#### Safety of the Pharmacy

A level of concern was raised by LRPOs, in regards to safety and security issues for pharmacies.

There may be a “higher prevalence of break-ins and people coming in demanding it. That would be a security issue that would need to be looked into.” (Interview #18)

As a result, despite the advocacy and encouragement, there was an acknowledgment of the ethical principle of professional autonomy and of the right of the pharmacist to conscientious objection, as long as a level of professional duty of care is preserved.

“I don’t think we can dictate that all pharmacists dispense it, because individuals may have their preferences and that is something we may have to accept. I just think we should encourage all pharmacists to be part of it and participate in it as they have a responsibility here to dispense these particular products.” (Interview #23)

### Stigma

#### Public

All participants identified the presence of public stigma associated with medicinal cannabis. Further to that, many proposed that the current illicit status of medicinal cannabis has led to this.

“Stigma will stay whilst ever it (cannabis) is illegal.” (Interview #19)

Many participants identified the lack of public awareness, influence of cultural upbringing, age and inability to distinguish between medicinal and recreational cannabis as key factors contributing to public stigma. It was apparent that most participants drew upon their previous experiences with patients on OST and opioid medicines, and would only be resolved with ongoing public health campaigns and further discussion.

“When you say the word cannabis people often just think about…the negative aspects and side effects.” (Interview #16)

“Imagining a place like Nimbin or you know a hippie crowd using it.” (Interview #1)

“Just from my observations, dealing with methadone patients…a lot of people look down on them and treat them badly.” (Interview #18)

For some, this public stigmatisation of medicinal cannabis was feared to affect the consumer and pharmacy/pharmacist rapport that they have established, with concerns that the pharmacy itself would be perceived in a negative light.

“In saying that, I don’t want my patients to think that we are a ‘cannabis pharmacy’ it might give us (franchise) a bad look in the community.” (Interview #27)

#### Media

Participants discussed how the media had played a predominant role in creating the awareness about medicinal cannabis whether negative or positive. Some suggested that the power of the media be harnessed for creating informed awareness on the therapeutic evidence behind medicinal cannabis and to dissolve negative stigma and rebuild the image of cannabis in a healthcare setting.

“You hear stories in the media… with some pushing for it to be legalised and others in the past highlighting it negative effects and this influences a lot of people.” (Interview #20)

Participants cited recent media attention advocating the medical use of cannabis, as a shift away from conventional views.

“I don't feel there is a lot of stigma to be honest. I think there's more like a push for it to be available. I've seen a lot of stories where it's been beneficial.” (Interview #12)

### Collaboration

#### Professional Training & Public Awareness

While pharmacists acknowledged a lack of extensive understanding about medicinal cannabis, it was deemed no different to any other new drug that enters the market. The majority of participants suggested the need for development of new training courses and learning opportunities, in order to ensure a greater understanding of the effects of medicinal cannabis.

“There will need to be education campaigns for pharmacists, consumers and probably all healthcare professionals around this issue when cannabis is legalised.” (Interview #23)

“Pharmacists have a great capacity… to learn and then disseminate information…to educate the public.” (Interview #31)

#### Unified Communication

Many participants suggested the development of a collaborative team of healthcare professionals to discuss the implications of legalising medicinal cannabis in order to ensure multidisciplinary care.

“There needs to be a forum where all key stakeholders who are involved in this issue…have a discussion. They need to raise issues that are going to affect them or their profession and considerations need to be made so I think that is very key.” (Interview #18)

## Discussion

This is the first study to investigate pharmacists’ views and concerns in relation to the prospective legalisation of medicinal cannabis in Australia, and one of the few in the world reporting pharmacists’ viewpoint on this issue. Given the global discussion about medicinal cannabis currently, there is a clear need to ensure that the views of all stakeholders involved are explored; particularly that of pharmacists, who have the role of medicine supply. Our study gathered the opinions of a reasonably heterogeneous sample of pharmacists, including professional leaders, and results indicated that a majority supported the legislation and decriminalisation of medicinal cannabis in order to provide a suitable treatment option to those with refractory and chronic medical conditions. Participating pharmacists described the need for suitable legislative and forensic frameworks that would allow legitimate supply under their scrutiny and recommended several models of supply. Pharmacists the world over, have demonstrated willingness and capacity for delivering harm minimisation services such as needle exchange programs and OST. Medicinal cannabis may be viewed in the same vein, where legal formulations dispensed with the purview of a trained and knowledgeable professional may be far safer than other means of procurement frustrated patients resort to. Pragmatic models offered by the participants and previous experience with pharmacy-delivered harm minimisation programs should drive future implementation programs for therapeutic cannabis provision. The perspective of pharmacists should also be probed in health systems considering legalising medicinal cannabis.

With no other study examining pharmacists’ perspectives to compare with, a comparison may be drawn with prescriber-based international studies. In contrast to our participants, the expressed opinions of prescribers worldwide have been relatively more sceptical with negative attitudes towards the use of cannabis medicinally. In 2005, a nationwide study exploring American physicians’ opinions about medicinal cannabis revealed that their level of positivity about legalised cannabis was lower than that of the American general public at that time [16]. In 2015 an Israeli study reported that 79% of Israeli physicians expressed cautionary views of support that medicinal cannabis “could be helpful for chronic and for terminally ill patients” [18]. As some researchers note, perhaps US physicians are not convinced of cannabis’s health benefits and believe its use carries risks [25]. Our participants did share these common concerns for patient safety but expressed that these are universal for all medicines, and for medicinal cannabis the benefits outweigh the risks primarily when needed to optimise quality of life in conditions recalcitrant to other treatments.

As illustrated in our study results, participants acknowledged the importance of continued training and learning in regards to information on medicinal cannabis. Similar needs for training preceding new program implementation have been expressed by pharmacists for other programs, such as the methadone program offered by pharmacies in many countries. In a 2001 survey conducted by Fleming et al. with North Ireland pharmacists, results demonstrated that pharmacists were willing to participate in methadone dispensing (OST) with a proviso that they be offered comprehensive training beforehand [26]. Pharmacists may not be unique in desiring training prior to supply program implementation. A recent Canadian study conducted with physicians highlighted their expressed need for “greater knowledge about…risks and dosing” before they could implement medicinal cannabis programs successfully [17]. Training is an essential element in implementing novel treatment. In a trial comprising pharmacists and opioid dependent consumers in Victoria, Australia, health policy researchers used seven key pillars to bridge the know-do gap in using buprenorphine for OST [27]. These pillars included skilled and experienced practitioners, government and policy support, incentives to prescribe the new treatment, specialist support services, clinical guidelines, training programs and patient involvement and information. Authors propose that this multi-faceted approach propelled the uptake of buprenorphine as maintenance therapy for opioid dependent patients in Victoria [27].

Our study also identified the need for greater collaboration to enhance transparency and involvement of all stakeholders, including pharmacists. This issue also appears to be identified in other studies; as collated data from surveys with Swiss pharmacists involved in methadone and needle exchange programs highlighted that “pharmacists do not feel integrated enough in the network of care of drug misusers and ask for better recognition of their role.” [28] Therefore from the perspective of practicing pharmacists, it would be important to establish an inter-professional forum to promote multidisciplinary discussion, collaboration and allow for consultation to take place across various disciplines. This, along with maintaining open lines of communication may help mitigate errors and ensure improved patient outcomes following the facilitation of medicinal cannabis [29, 30]. Successful specialised treatment programs, for example buprenorphine provision, have often utilised inter-professional training for pharmacists and specialist physicians [31, 32]. Similar methods need to be applied in the case of medicinal cannabis as well.

A major issue emergent from our study was the need for nationalised legislation to maintain uniform regulatory policies. This view is reflective of international literature that documents the long-running battle between federal and state law. A US article contained a Californian Supreme Court ruling in 2005 indicating that “patients who take cannabis in states where its medicinal use is legal are not shielded from federal prosecution.”[33] In 2008 a survey study by Reiman et al. reported medicinal cannabis facilities followed a “social model of care” with patients creating a system of self-dispensing without professional intervention as a result of lacking legislative mandates on medicinal cannabis supply [34]. In order to prevent cases such as these and to increase pharmacists’ confidence, our study highlighted nationalised legislation as a key driver to successfully introducing medicinal cannabis into Australian healthcare.

Participants in our study also identified safety as a major area of concern.

For reasons of safety and reproducibility, introducing standardized forms of synthetic or extracted cannabinoids supply, although more expensive, rather than extractions of phytocannabinoids in the cannabis sativa plant, would be ideal. However, there are no globally recognised standardized forms or formulations of medicinal cannabis available yet, due to vast variability in constituents from plant to plant. This could be a future development once medicinal cannabis is legalised and research/development is allowed to evolve. Suggestions also included pharmacovigilance to monitor patient safety, addressing risk of abuse and creating support to reduce prescribing under duress as well as establishing robust support for pharmacy safety. Such suggestions were drawn from past experiences with OST and the supply of dangerous drugs of addiction. A 2010 survey study by Winstock et al. reported pharmacists’ had experienced problems with OST clients showcasing “aggression, intoxication and remaining in debt with the pharmacy” [35] Indeed, increasing reports of pharmacies being robbed or held-up are noticed globally [36, 37]. And for pharmacies to supply medicinal cannabis this needs to be specifically addressed.

Stigma was identified as a major barrier that needed to be mitigated in order for medicinal cannabis to be successfully rolled out. The influence of negative stigma on the actual adoption of such medicines and health programs is revealed in various studies. Comparably our participants described the stigma around medicinal cannabis paralleled that enveloping methadone clients. Another recent qualitative Australian study exploring pharmacists’ opinions about methadone supply as an OST, reported that stigma appeared to be an underlying cause in the case where pharmacies were still unwilling to provide OST despite increasing demand. It reported these participants showcasing negative attitudes of “prejudice, cynicism and fear.” [38] In our study, it was recognised that stigma may be a result of the blurred lines between medicinal and recreational use of cannabis. To shift public opinion, greater awareness and education needs to be implemented, in which clear distinctions between the two uses of cannabis are defined.

All of these themes were triangulated to generate a conceptual framework (Fig 1), which captures the results of the study and showcases socio-political drivers for the legalisation and supply of medicinal cannabis through community pharmacies. It is a conceptual map of themes emerging from ideas expressed by participants in this study. The conceptual map identifies secondary drivers, such as “safety of patient and pharmacy” for example, which together formulate primary drivers of change. The four major drivers of change in this particular case were identified as: 1. Addressing Safety, 2. Drawing up inclusive legislation reflecting all stakeholders’ concerns, 3. Addressing Stigma and 4. Establishing suitable collaborations.

This conceptual framework can be utilised by policy-makers to develop policies of change based on primary sourced data of pharmacists’ perspectives.

## Limitations

Some limitations of this study include the diversity of participants interviewed, which were primarily from a community pharmacy setting, and this is not generalizable to the entire pharmacy profession. Despite interviewing participants from various locations across Australian a broader location sampling would provide more widespread and rigorous results. Video recording of the interviews may have also strengthened the study through paralinguistic analysis of captured non-verbal cues.

## Conclusion

Findings of this study (S1–S5 Files) highlighted the perspective of pharmacists who hold the important role of suppling medicinal cannabis, thereby identifying important factors which can help shape future policies for the successful implementation of medicinal cannabis in healthcare. We recommend that these views and strategies be incorporated in the development of new policies and legislations.

## Supporting Information

S1 File Barrier and Facilitator Quotes. (DOCX)

S2 File Legislation-Regulation theme Quotes. (DOCX)

S3 File Role of Pharmacist—Collaboration Quotes. (DOCX)

S4 File Safety—Product Standardisation—Formulation theme Quotes. (DOCX)

S5 File Stigma theme Quotes. (DOCX)
