# Supplementary material for: The Role of Medicinal Cannabis in Clinical Therapy: Pharmacists' Perspectives
Source: PLoS One. 2016 May 12;11(5):e0155113. doi: 10.1371/journal.pone.0155113 (PMC4865212; doi:10.1371/journal.pone.0155113)
Supplement: S1 File — (DOCX) [file pone.0155113.s001.docx]

| **QUOTES** | | | |
| --- | --- | --- | --- |
| **Comment** | **Barriers (-)** | **Comment** | **Facilitators (+)** |
|  | The Opioid Substition Program (OSP) that kind of style but then it can’t be too difficult as well because these people are incapacitated to an extent as well, so I think there will definitely be some issues around that to be dealt with. |  | I mean I just take as if it's going to be like any other S8 drug and it would be best suited in a community pharmacy setting for access reasons. |
|  | accessibility has got to be a big issue, different times to have access to it, what about rural areas, so I think there will be a role to play with it |  | We should try and give access to the people that really need it because, I mean, if there is can ultimately be positive affects as long as we do what ever we can then we might as well give these people the opportunity to uhm to use these medications appropriately. |
|  | Obviously the more stringent the laws are and the implementation the less issues we'll have with it. Dispensing it more freely, sort of it's just thrown out there, then we're going to have issues with patients coming in and wanting abuse it, just like we have with our oxycontins. |  | I guess, the community pharmacist can really be an advocate for it and be telling people other options regarding their condition. Because most people would be in contact with their community pharmacist, easy access. |
|  | my concern it would be being able to get in contact with the doctor because if it's going to be used for certain things that are quite common like epilepsy and things like that, not like pain, well where do we go from there? So what can I offer these patients if I can't get in contact with the doctor? If I can't verify a prescription, things like that? |  | It's the accessibility to the drug, you know what I mean? It's all about accessibility. Community pharmacies are all over. |
|  | I think it would be better if it was done as an authority script because we know it reduces the amount of forgery and things like that because it's hard to forge an authority script. If it doesn't go through we know at least a doctor's been behind it. |  | It needs to be available to people that are suffering |
|  | I think the only concern is if it gets into the wrong hands and people start abusing it. That's where we, from the beginning, need to make these laws and make sure that these laws are as strict as possible. |  | in a community pharmacy because of accessibility so you always want to just make it easier for the patient and I think if it’s just in a hospital or just in a clinic I think that can make it a bit more difficult so having it more accessible is really important. |
|  | even with S8 medications, just read at article just a few day ago it's usage has like sky rocketed in recent uhm years which is obviously uhm quite worrying. You know that prescription medications are being abused more heavily like in recent years so there is a potential for this as well to be misused. |  | hospital setting would be too limiting for patients to access and same as clinics, I don’t think clinics would be widely spread enough to make them convenient enough for patients |
|  | I worry about is you put this in a spray, in a liquid form and it's easier to sort of abuse if you think of. Uhm so ideally, for instance with oxycontins you have the new formulation so when a patient try's to sort of use it illicitly, it's very, very hard just like also suboxone, suboxone containing component. So I think that would be a good way to sort of prevent diversion is by putting it into a formulation like that with the science behind it and to make it hard to abuse. |  | That's our role. We are health professionals and if we identify abuse then we refer people to the correct uhm avenues. That's what our role is as a pharmacist. We are not here to make people who have a problem with medicines feel like they're criminals and we refer them to the appropriate health facility. That's we do the Needle Exchange program. |
|  | I think it could be abused. I just don't think you can trust all pharmacists or just the studying, I just think it's specialized and it shouldn't, it needs more, it probably needs like, you know, pharmacists and a specialized team |  | you would be naive to think there wouldn't be any challenges associated with implementing medicinal cannabis but that is where pharmacy is great at developing key strategies which we can then apply to other practicies. |
|  | Do I even know this patient? Have they been here before? Are they doctor shopping? All of the same things you'd see with an S8 prescription. You know you do get funny circumstances that will arise with S8 prescriptions so it's hard to say. But I think we would still see the odd person who would use it for recreational purposes come in with a fake prescription for cannabis. |  | you know we deal with these substances every day. You know we have opioids. We have methadone. We have, you know, ketamine, you know, we deal with things, illicit substances every day and we do it well you know. |
|  | The only thing I can think of is they could change it like the oxycontin new formulation in which case if you try to crush it, whatever, you can't physically do it in which case they wouldn't be able to abuse it. |  | So I think, yeah, there's a chances for it to be abused but I think community pharmacy, you know, knows what’s best and is ready for it? |
|  | so there would be something that happened that would be similar to the DD or S8 drugs yeah, that would be similar, you probably get like forged scripts and that would be because some of the Doctors that they prescribe the medication for the patients there will be more afraid to get the scripts for them. |  | I think that the patients, from our understanding who'll be on this, there's not an abuse issue at all. It's just using it for the medicinal purposes which have been proved beneficial. |
|  | yeah why not be in a community setting but maybe for now, to get used to it all, how people will react to it, maybe we need to settle for a hospital setting with doctors around. It just depends on how the trials and how people have dealt with it and things like that. |  | I think they'll be cases where people who are abusing it and so they're not using it for the right purposes and there might be challenges with that but it's no more than dealing with, I don't know, like people who are addicted to oxycontins or something like that. |
|  | This is pretty new ground for pharmacies if this was to come around, especially in community settings. I suppose I hadn't thought about it too much about what they would do, like in a community setting |  | if the laws are in place that cannabis can only be prescribed by a specialist and with this particular authority and that authority is, you know, is linked into Medicare so we can prove that is legitimate and we can see the indication that has been written for that patient I think the potential for abuse is a lot less. You know what I mean? It'll just be the way they regulate it. |
|  | but if it was to be imposed I think that definitely it would need to be under very strict conditions and strict guidelines and everything. |  | I don’t know whether it would be abused because it’s already available in the community, it’s not legal but it’s still happening, so it may or may not be abused it would depends on the dosage and whether it can be converted into an injection so a couple of things come into play. |
|  | they're on a special kind of special program, a special listing just like what we do with S8 medications but I suppose even more stringent control measures so that it's used can be uhm limited to them and also be supervised. |  | obviously there is the potential for abuse with this medication but that goes with a lot of other medications as well, so you pretty much treat it like anything else, so yeah as long as there is proper regulation all that sort of stuff, you will experience problems but it’s just like any other medication and a lot of programs that are out there as well. |
|  | we're missing is just solid evidence and solid information because there's a lot of misconceptions around the actual substance itself. |  | I think it’s quite separate because it’s already widely and readily available and cheaply available. So I don’t think diversion would be as big an issue as it is with a lot of other Schedule 8’s. |
|  | And if someone does have something against mediicnal marijuana it would stem more so from an upbringing and cultural thing  No not really. I don't think I play a big part in it at the moment although I guess it depends, I guess, depends. Like I've heard stories of it being like a what you would call a marijuana clinics in America and so there's people who have to, in charge or in charge of these clinics and people who have to oversee the use of it and perhaps one day that'll be a part of pharmacy but I don't see that happening any time soon so. |  | skimming the media, there doesn't appear to be a lot of resistance from the medical field towards it as well. So I guess pharmacy really hasn't had a voice because they're quite happy with it. I think more pharmacists will take it on board. I mean there's always an ethical, they can always make an ethical decision whether they want to keep it within the pharmacy or not but from my industry I really haven't heard much resistance for it. And I guess within the medical field there seems to be an overwhelming support for it. Perhaps a little bit of resistance but not a lot. |
|  | I don't quite honestly think that we have to do too much apart from counselling and learning about it and learn about it's properties and side effects and stuff like that. The it's up to other people to determine if it's good for the public. |  | I guess, the community pharmacist can really be an advocate for it and be telling people other options regarding their condition. Because most people would be in contact with their community pharmacist, easy access. |
|  | I can’t explain like clearly why but I am thinking if it’s been legalised there is an attempt it is easier than before to be abused. |  | I think if they do it properly it has got to happen, |
|  | Interviewer: Okay sure, I guess hypothetically speaking if it were to be legalised would you be happy to dispense it, if it was legalised or not so much?  Interviewee: Not so much. |  | People who are using it are perhaps not under medical jurisdiction when they use it, because it’s not legal. And probably it would be far more safer and far more effective for it to be made legal rather than the current situation. |
|  | Let the Pharmacy Department in the Hospital that they can dispense it but not like the community pharmacy to dispense it. |  | And I think that's where uhm it becoming legalized is really, really important. One it takes the stigma away from it but two it ensures that patients are getting the therapeutic dose that they require and then not playing doctors or pharmacists themselves, but they actually have a health care team behind them. |
|  | there’s the issue of stigma, there's the issue of having adequately trained staff, there's the issue of making sure that all the protocols for dispensing it are followed and maintained. |  | it's just another chemical. I guess, I'm not 100% sure about the long term side effects or the certain side effects of using it for such a long time but there's no drug out there that does not have a side effect. Every drug has a side effect because it's going to be working somehow. And obviously if you overdose or if the dosage is a little bit too high you will get side effects. So I don't really think there's much problems with using medicines like this one. |
|  | it's related to people even getting like schizophrenia and all these different things as well as far as I know. |  | And in relations to cannabis I don’t think it would, I don’t think it’s an expensive enough drug to make it a risk, a security risk for pharmacies. I don’t think it would ever be like the old days when people held up pharmacies for their pseudoephedrine or methadone I just don’t think cannabis is expensive enough. |
|  | just for safety as well of the pharmacy and the pharmacist, it should be stored in a safe where people don't have access to it |  | it’s a natural product it’s something that is relatively cheap and with the rate of discovering new medicines it costs a lot particularly for the government and hence the tax payer in regards to economic sustainability. And cannabis is a medicine that we have had access to all along and it can benefit so many people in paint, palliation and epilepsy |
|  | also the risk, also teenagers and the risk for the future of psychosis, |  | already widely and readily available and cheaply available |
|  | with recreational there are short term and long term side effects that are known, so the psycho-active side effects and things like that and so you know it has been quite harmful to a lot of people and that’s why it’s considered illegal, |  | it seems to be an overwhelming evidence as well as hearsay of people who have used and a lot of medical practitioners who've, in a way, recommended it as a last resort and how great it's been. |
|  | I certainly think its much better than legalising it full stop (both recreationally and medicinally) you know, that is really dangerous because it is a very dangerous drug you have got to remember that, it’s one of the hardest drugs to get off, from what I understand I had a brother who was on it and it’s made him schizophrenic, all sorts of stuff, people see it as a mild drug getting off it is supposedly harder than a lot of other drugs, |  | any pharmacist operates on the guides of evidence based practise and obviously there will be some pharmacist that will hold moral or ethical objection to supplying, that is with anything. But the vast majority of the profession are evidence based professionals. And yeh as pharmacist we are going to have a massive role in supply and education. So we should have a big voice and a say in how its implemented. But in saying that I would want to ensure it is the right people from the industry being involved in that debate. |
|  | and I think for like a community aspect as well in terms of safety, would that result in a higher, higher prevalence of you know break ins and you know people coming in demanding there might be a security issue |  | it has a case in therapy where we have seen in overseas models it does have, it has a place to play, you know in paliative care and pain management which is something we recently did a project with Joyce Swan who is a chronic pain specialist we had a little discussion about cannabis use in chronic pain management when traditional therapies dont work and I think there’s an area for it there, so I definitely think it has alot of opportunities. |
|  | I have got lots of experience. We’ve got a lot of mental health patients who’ve got a really strong relationship with the assertive out reach team out at royal north shore hospital and we deal with lots and lots of their schizophrenic patients and a whole lot had their schizophrenia appear when they were adolescent and heavy pot smoking adolescent males. |  | I don’t see why medicinal cannabis shouldn’t be available to those patients who have tried you know first line, second line, third line treatment and you know so that they can have an improved quality of life, who are we to restrict them of that opportunity, so I think you know more research needs to be done and I need to know more about the evidence as well |
|  | importance of waiting for evidence to have assurance both for patients and suppliers that the supply of cannabis is standardised and of therapeutic quality, even if it means waiting it out. |  | it's really important that they voice their opinion. I know that the previous and the current uhm, particularly the previous health minister and also Mike Baird talk about legalizing it, particularly in New South Wales, but then also using pharmacy as a channel. |
|  | To be honest with you I don't, I'm not against it. I'm all for it if you've got all these evidence for me showing that it actually works. |  | Today I guess what social media has been good is its supply of information for evidence but at the same time it does contribute to alot of misinformation and its I guess healthcare professionals or educators to kind of filter that out as well. |
|  | I think with all types of prescribing especially prescribing narcotic medication, there needs to be strict evidence based guidelines, which need to be adhered to and obviously the prescriber needs to be appropriate within those situations |  | just recently I have seen the topic brought up just in the media, but I know that it has recently been legalised in America in the United States of America obviously for the medicinal purpose so that’s the thing about the media coverage with that, that has created interest amongst myself, personally I don’t know much about it, but I did see a story relating to an Australian girl that was suffering some sort of condition which resulted in her being in a lot of pain and this girl had tried a lot of things and I think the University of Sydney is actually involved with this case from what I was watching I think there was, I think the parents were trying to sort of get try medicinal cannabis as a treatment option |
|  | well as long as it’s proven there should be a place in therapy for it and as an alternative to a lot of others that are pain relative, but I think until that is proven, I don’t know, I guess you got to get the cart before the horse, the horse before the cart sorry. |  | we're in the field of medicine and helping people and if a certain medicine, no matter what it is, is help the quality of life of a patient therapeutically then there should be no issues or problems with it. Uhm for me, speaking from experience, if a certain medication could have prolonged or made a better quality of life for a cancer patient, you know, that is a big thing a patient that, it can help with their appetites and things like that and that's one of their biggest things that they have to struggle with |
|  | it's heard on the news and it's usually involving he police and, you know, big heists and all sorts of criminal offenses regarding the substance itself. That's the reason why we have that sort of stigma cause all you hear about it is the bad stuff because it's not legal. I guess it's more connected to criminal activity and abuse rather than medicinal things here. |  | I'd have to understand a little bit more about the pharmacology behind it because... you know... is it better that it doesn't pass through, first pass metabolism? Is it more effective or, you know, is the efficacy far better in that case and the side affects less? You know, I'm not sure how much the gut would break it down in terms of a capsule form, inherently, you know, I guess there's a reason why people smoke cannabis. And perhaps that's the way that it's more effective. I'm not sure. It would be interesting to know the pharmacology behind it and see which one's better. Perhaps, you know, there's ways it could be a vapor that people inhale. |
|  | That it’s hearsay a lot of people because it’s out there more and you know you get those stories where it kind of "I saved my daughter with cannabis" that kind of thing so I think it is just more vocal. |  | I would imagine that if a scheme for making legal Cannabis available was put through community or through even specialized clinics, it would be along the lines of something like methadone clinics where it’s dispensed, it’s recorded, there’s a special indication and people are being monitored by a specialist |
|  | we need to give the community and the government you know the support and uhm the reassurance that we're supportive of this as well. Otherwise you know if there's no support from us as well then there's going to be more barriers in terms of trying to legalize this and to implement this as well. So it is important as well and even from the patient's perspective as well they need to understand that, you know, we're supportive of this and we don't look at this in a negative way, we look at this in a positive light as well, and that they're using this for a legitimate uhm therapeutic purpose then I think definitely our role is quite important as well. Everyone, I suppose, has different roles and we all complement each other. |  | So just like heroin is illegal, methadone is not an illegal substance when used in the pharmacy and used appropriately and being monitored and the scheduling of it and things like that and I think we should use the same template for medicinal cannabis. |
|  | there is so many flaws in the quality and safety of the substance that is taken regarless and thats because tehre are no controls or regulation on it supply. It's making the wrong people rich and is providing an avenue for potentially ruining the lives of young mainly men that take it upon themselves to supply it. |  | I don't think medicinal cannabis should be like the methadone program. Okay so I think that that's also an area where people are thinking like dose on a daily basis or have that sort of really strict protocols and I'm not sure that's appropriate for that [medicinal cannabis] use because I think that the patients, from our understanding who'll be on this, there's not an abuse issue at all. It's just using it for the medicinal purposes which have been proved beneficial. |
|  | Well I think cannabis should be the exact same. If a patient or somebody is found with it without a prescription, without authority, that's when deemed illegal. Whereas if the patient has the authority from a doctor, has a valid reason to be using it, then I feel in that case it's medicinal. It should be legal. |  | it'd have to be monitored intensively by possibly a pain specialist not just any regular GP or a person that uses it here and there. I has to be properly managed. |
|  | I think it should be legalised medicinally, I don’t think it should be legalised recreationally but I think it needs to be highly regulated for medicinal and there will have to be sort of appropriate auditing processes and things like that and that continue that don’t sort of lapse after the first few years so of the program being implemented so, we know things like the Opioid Substition Program (OSP) that kind of style but then it can’t be too difficult as well because these people are incapacitated to an extent as well, so I think there will definitely be some issues around that to be dealt with. |  | like to see pharmacists be heavily involved in the supply of it, for providing the information and also in its regulation of its supply.  One of the things that has held pharmacy back in the past would be the absence of a national register or national database, and I guess establishing a nationalised system and accompanying that with the current E-Health Scripts that would help manage this. |
|  | should be S8 even incorporating aspects of maybe the clozapine programs and the OSP in terms of recording so whether it’s an online portal like for clozapine but umm regulations associated with dispensing and things like that. |  | S8 I think that gives the pharmacist who is giving the medication, some comfort about the level of uhm legislation behind them checking the medication, you know, make sure the doctor's write the prescriptions properly |
|  | It’s like the emergency contraceptive pill you know some people are okay some aren’t, in my opinion I would be as long as it’s done the correct way and you know it’s not offered to everyone and you know just like oxycontin which is abused, it would have be to regulated. |  | like to see pharmacists be heavily involved in the supply of it, for providing the information and also in its regulation of its supply. |
|  | visible in terms of doctor shopping it would have to be, that will have to come into play and also I think with the digesic regulations where the Doctor needs to provide written authorisation where the pharmacy needs to keep it in a pharmacy for a certain amount of time. |  | And it's regulated in terms of uhm who's actually able to prescribe it and not any general GP can prescribe it but rather a specialist in a particular area and someone who's done some specific training in that area. |
|  | it depends on who is going to be able to do it, what the rules are, what’s the cost I suppose, all those sort of things, there is a lot of things for the Government to sort out. |  | as long as pharmacists are aware of the legalities behind it so perhaps there is a specific training for pharmacists to be able to, for example with our QCPP. Say if there's something integrating with that in terms of the pharmacist's procedures and they follow procedures around in cannabis prescriptions and that it is locked in a safe and it's monitored |
|  | I believe it should be legal, definitely, but with a lot of terms and conditions. |  | if the laws are in place that cannabis can only be prescribed by a specialist and with this particular authority and that authority is, you know, is linked into Medicare so we can prove that is legitimate and we can see the indication that has been written for that patient I think the potential for abuse is a lot less. You know what I mean? It'll just be the way they regulate it. |
|  | But its when its treated as a criminal issue not a health issue thats were its becomes an issue. Say when it becomes a health issue it would be so much easier to deal with this,these people.... like for the wider society. |  | I think that they should be decriminalized and regulated and then if people have a problem, as in addition, made criminal just like it does with alcohol. |
|  | At the moment what they're talking about it's going to be so restrictive |  | I don't see why it would need to be a Schedule 8 medicine. Schedule 4 would be fine. |
|  | (coming from a reasonably uninformed position) I’m not convinced that it’s necessary I assume it’s going to be used for nausea for terminal illness and maybe some pain relief, its indications. But I’m not convinced that there aren’t just as effective products that are already available |  | Probably like an schedule 8 I would think.  Or you know like the, I'm not too sure but is it clozapine, like they have like a program where they have to keep in touch with someone, something like that maybe? |
|  | So they're all smoking. So obviously that might not be the dosage form that they're going to choose. Simply being maybe a tablet for a different sort of form. |  | I don't think I see it as a S8 based on its use to be honest. I don’t really see it as the same thing as methadone really. I see it more as a schedule 4 maybe recordable. |
|  | even if they changed the formulation I don't think that it would, I guess, improve these problems that we would encounter. The only thing I can think of is they could change it like the oxycontin new formulation in which case if you try to crush it, whatever, you can't physically do it in which case they wouldn't be able to abuse it. |  | No I just think it should be done and it’s got to be a national approach obviously the PBS is run nationally so it’s going to have to be a national, there’s no use throughout Australia saying yes and you know whatever, it’s got to be national and something that is agreed on and is done and implemented |
|  | Especially when you get it from the black market and things like that, you might not even get pure substances, so getting synthetic forms of it can be way more dangerous than the natural ones, so I think getting the right medication, the right form of it |  | I think private use, it should be decriminalized that’s how I feel about it, because I think it’s a absolute massive waste of resources booking people for smoking a joint, that deserves to be so far away from their criminal justice system. It’s not funny it’s just a huge waste of time, So I can see how with mental health, I can see where decriminalising and has its risks so I think decriminalising it and education campaign, not to dissimilar to what Portugal has done. But stop wasting time on making it as if they are badies they aren't badies they are just less risk averse. |
|  | I think it should remain illegal. |  | Like I think once if its established that it’s not abuseable, there is no reason why it shouldn’t just go into an S4 category until its safety is well established and maybe one day if it’s a harmless antinausiant, prochloperazine is now available over the counter and like I would have absolutely no problem recommending a cannabis spray. Once the safety is been established. |
|  | Look I think any formulation will be fine, as far as dosage goes I have no idea what would be an appropriate dosage or not, dosage forms though I suppose anything other than smoking would make it seem more medicinal less recreational. |  | strategic thought given to the channels for how it would be available to patients. I believe that actually making it legal is maximising its utility. we need to have evidence from very good clinical trials and once that’s there, it would actually be put into the registers by TGA or the authorities |
|  | But overall I definitely feel pharmacists are undervalued. Studying for five years and then being payed only $1-$2 more than a hospitality worker is not right for a health care professioanl with so much responsibility and who continues lifelong education and accreditation is just a joke. |  | it shouldn’t be in the hands of a policeman to decide whether it’s recreational or being medicinally used or both. |
|  | You're going to have the division, they'll be divided. But as long as uhm a pharmacists does not have to supply it he's able to direct patients where they can get it into pharmacies. they're not completely against it's use and therapy. I think it's the pharmacist duty to care, to direct the patient to a place where they can actually get it because it's not their decision as to what the patient gets. |  | anything that can help someone prolong their life or help their quality of life there's no issues, especially if they're being monitored by a health professional and things like that, there shouldn't be any concerns. |
|  | We believe that the medicine, the cannabis use needs to be treated as a health problem not a criminal problem. |  | just like any other medication. You know uhm if it has a positive affect then why not? We might as well use it's positive affect if it's going to help people out then why not? I mean that's our role is to help improve people's uhm well being and obviously quality of life as well and why not? That's what I think. |
|  | I just think it's specialized and it shouldn't, it needs more, it probably needs like, you know, pharmacists and a specialized team rather than, I just think a pharmacist now are already, they have a lot of duties and stress enough. I just think you kind of need, it's sort of like doing methadone. It's kind of like a separate thing so I think it might be too much. |  | medicinal cannabis should be legalized in Australia and I think that would be great to see Australia being one of the first countries to do that. I know we're not the first but it's certainly being within that the first uhm mover. I think that it will help a lot of people and be beneficial to a lot of people. And I think it's very appropriate to utilize the pharmacy channel. |
|  | I would say it’s not important at this point in time, because as far as I am aware there isn’t enough evidence based information readily available for us to make an educated decision, as far as I am aware it may be out there but I am not aware of it. |  | it should have been introduced long ago, the whole basis of Cannabis staying legal, is a bit of a farce, I certainly believe in medicinal cannabis |
|  | the pharmacist has to be compensated for doing it, I presume, I don’t know how they are going to do it you know through the PBS or whatever, |  | committee should recommend a standard formulation containing those moiety be prepared and then that’s the one standard formulation that’s made available nationally. |
|  | I don’t want to be in a position where all of a sudden it’s legalised, I have got patients coming into my pharmacy going yeah I am not prepared that being the worst case scenario, I want to make sure all the professional organisations do communicate to the profession effectively and you know give us enough time to really grasp this because we can’t get it wrong we need to be you know prepared for this |  | it ensures that patients are getting the therapeutic dose that they require and then not playing doctors or pharmacists themselves, but they actually have a health care team behind them. |
|  | And to me that’s absolutely the way it should be it’s pointless chopping and changing and doing things. Definitely it’s probably stronger if it’s a united voice anyway or some kind of collaboration before we submit the same thing. I would like to see pharmacist given again more responsibility because I trust them. |  | I think in the compounding pharmacy setting it would tend to be the actual alkaline. You wouldn't be giving the plant because you need to have a regulated, the whole idea of regulation is that you had to get the correct dose. |
|  | always imagining a place like Nibin or you know a hippie crowd using it |  | As long as the active ingredients are produced or packaged by a TGA licensed facility then I don't see why there'd be a problem. |
|  | there is a level of stigma, media wise and public labelling wise |  | I don't think I would mind any form I think with like a set dose or a set sort of measurement that you can't adjust, the difficulty would be where it is prescribed in different quantities... so as long as it’s a flexible sort of form I don’t mind what it is. |
|  | The overcasting or the shadowing of its potential for good use by its recreational use and the bad side effects of that is what's affected progress in this area. |  | if you look the DD Cabinet now you have got all sorts of forms you have got patches, you have got tablets you have got suppositories, you have got you know, we have got sprays on the shelves, whatever form works the best I suppose, I don’t see that as an issue. |
|  | the name would not be cannabis so maybe would lose some of the stigma that's attached to it if we loose that name and give it a medicinal term and educate people that it isn't in fact cannabis in the way that we know it. It's been purified. |  | So liquid form, from my point of view like that would be more versatile in different demographics, different age groups, so it’s more, obviously as I said it has to be evidenced and I can’t really I can’t really comment on what’s appropriate and what’s not because I am not in a position to, but what I can say is that it needs to be that evidenced in order to which form of administration would provide the best outcome for the patient, |
|  | Lack of education mainly and without educating the patient as to when we say we want to make cannabis legal if obviously it's on the news, "Do you want to make cannabis legal or not?" It's a bit like, "Ahh yes... I don't get it... everyone can smoke marijuana and grow it in the back yard." That's the first thought that comes to a general person's mind who's not a pharmacist or maybe even if they're a pharmacist. |  | It's quite exciting. There's finally going to be a treatment option for those that up until now had no hope and no treatment. And as a pharmacist I guess the patients, you know, health and well being is at the upmost of our priority and for us to be able to help these patients and especially children, to give them a better quality of life and to improve on that is something that basically we work for every day. |
|  | if people don't understand that maybe they should go and see a part of the care, a hospital, and see what these people are going through and then make that decision if they are quick to judge. If that's the case then further education, even for our pharmacists or our health professionals is needed. |  | We still have an important role to play. I think ultimately, of course, the prescriber, you know the doctor and the specialists, I think would be playing the major I'd assume. But we still do play an important role as well because especially as advocates to something like this if they were to legalize this I think we do still have like an important role as well. |
|  | That stigma is pretty much dying now in terms of it's usage. I'm not saying there isn't, there is definitely a stigma but it's stigma is becoming less and less prominent in todays society because of this potential you know positive affects. |  | we're all pharmacists and so we know the therapeutic benefits of it. You know, we can read the evidence and, you know, we can understand how it works. So I think, yeah we should uhm, we should have a voice. |
|  | I think there's always going to be a stigma attached with that. I think as soon it becomes uhm you can use it for therapeutic treatment and the evidence behind that and I'm sure there'll be a public awareness campaign behind that. I think the stigma will be reduced, you know, substantially. I think cannabis is milder drug than the other illicit drugs that are on the market as well. |  | cannabis is going to be legalized and it's going to be brought to pharmacy, community pharmacists need to have a say because not all pharmacists are going happy to dispense it and also they should feel involved because really they're going to be providing the access to the patients. I think it's very important that pharmacists actually have their say because they're providing the drug. |
|  | it's heard on the news and it's usually involving he police and, you know, big heists and all sorts of criminal offenses regarding the substance itself. That's the reason why we have that sort of stigma cause all you hear about it is the bad stuff because it's not legal. I guess it's more connected to criminal activity and abuse rather than medicinal things here. |  | do you think it's important that pharmacists speak in this particular debate or do you not see it as their place?  Interviewee: Of course they do if they're going to be involved. |
|  | And if someone does have something against mediicnal marijuana it would stem more so from an upbringing and cultural thing ...its a massive generalisation i know but it is seen in a wide range of issues and people don’t want to accept something new, different or a change. |  | of course because we get a lot of first hand info from patients or customers. You know they come and tell you their stories and what's working for them and what isn't working for them. The doctor was too rushed. Didn't have time to listen to me type of thing you know. It seems like their first port of call is the pharmacy now, their local pharmacy where they want a solution and free advice. |
|  | I would say people mainly are against it because it is illegal and they don't want to break the law. |  | Yeah I think the pharmacies voice is important because they will be the ones administering it and having to monitor its use and that kind of thing, so I think they should definetly voice that they are for it. But it should be with the appropriate systems and legislation in place and all members of the healthcare team should be involved so it shouldn’t be just you know hospitals or doctors as they are the ones that can do it, it should be you know doctors are trained to prescribe in a certain way then supply it within the community or hospitals and these are the procedures involved in that and aftercare is involved in that as well, so I think it should be continuous. |
|  | we pharmacists need to be a lot more up front about the health issues involved with any sort of drug addiction and that we need to be the ones that then refer people on and not be judging people. |  | Yes I think it’s important what they should be saying is really up to the individual pharmacist and what they beleive in, but personally I support it and it’s done in the correct way and it’s regulated it’s a Schedule 8 there is no risk, in my opinion. |
|  | if the general public have a negative view on it it would be more because of lack of understanding or experience or that don't know anyone that's been in that situation or even had like terminal illnesses |  | Yes if they feel strongly about it and if they are well informed of the trials and the benefits of medicinal cannabis then I think they should because it’s just a step forward and it will help a lot of people and you know a lot of people they try and seek their medication for medicinal purposes but because it’s illegal it makes it very difficult for them and they are up for criminal penalties and things like that so you know sometimes they are there for a general reason and so if you can make that easier for them, if it’s legalised that would help a lot of patients out, yeah I would definitely encourage people to you know step up and support the program, if they can, yeah I think it would be, it’s good progress. |
|  | The media really you know play it out to be soley an illicit drug and you see all those stories of plants being grown and discovered in rural areas and use for personal gain and sold on the black market and even areas like Nimbin which its almost legalised and so there’s pockets around Australia where it’s almost ignored by the Police and authorities but that’s again that might not be the case, but that’s how I perceive it. |  | if we have a role in that then that’s huge. |
|  | I think it does have a lot of negative connotations in the community, so often when you say the word cannabis people often just think about you know the negative aspects and side effects. |  | it will be interesting, but I can’t think of a better place to do it, any pharmacy really should be able to do it, should probably have some pharmacists that might not want to do it but yeah I don’t know, it depends how they are going to regulate it |
|  | not educated enough about it so they may still have that stigma about it because time goes on and as you know progress occurs with it, I think they will be more informed and I think the stigma will come down, but I do think it’s definitely still there. |  | Seriously I am more than happy to dispense it, well as long as it’s really clear, the rules and regulations, as long as we think we are doing some good, I mean what’s the Doctor’s moto I think that’s what our moto basically is as well, first do no harm. |
|  | there is stigma with it, so anyone you know I know that anyone uses drugs especially illicit drugs you know society looks down on them it’s just the way it is, not everyone is like that, I can’t generalise it, just from my observations because obviously dealing with these methadone patients, so I know that a lot of people look down on them and treat them badly, we don’t really know their situation and a lot of people jump to and they don’t know how these people got into these situations in the first place, so there is a stigma |  | Well I think it’s very important if they are going to part of the delivery process, yeah definitely, if we are not then it probably doesn’t matter but I would have thought the only way of making it happen, you wouldn’t want to have shops like they do in Colorado you know just giving it out willy nilly, I think Australia is not looking at doing that, for the right reason, I think the obvious thing is that pharmacy is the place to deliver it I would have thought. |
|  | I think our generation, younger generation are becoming more accepting and are not so black and white, we see the grey areas more and yes these things can have potential in a sort of medical treatment, rather than just recreational use, but I think the older generation may view it differently, so yeah that would be my view on it, yes there is stigma. |  | Who else can do it, no one else can do it, Doctor’s I don’t think would want to do it, I just think no one else can do it really other than pharmacists. |
|  | Oh I think it is the fact that it’s illegal I mean the stigma will stay whilst ever it is illegal. |  | Because it’s going to involve pharmacy whether we like it or not, because you know we don’t know how it is going to be supplied but let’s say it is supplied through community or hospital you know it’s going to involve a pharmacist and you know because it will involve us, we need to have our input into that matter I think that is very important you know we are most likely the ones dispensing supply |
|  | I absolutely believe that there is stigma associated with it. I think pharmacist see cannabis as being a Central Nervous Depression with a psychoactive component, and whereas consumers I think just see it as something to get high with. |  | there are a lot of other issues to think about with this and implications which are not necessarily medical in nature, that do affect pharmacists so you know a lot of things, so we need to have our input, there needs to be a forum where all key stakeholders who are involved in this issue need to have a discussion and they need to raise issues that are going to affect them or their profession and consideration needs to be made so I think that’s very key, I think that is something that should not be rushed into, so that would be my thought on that. |
|  | So I think Australian consumer’s perception of what's going in North America is that they’ve just opened a whole lot of dope shops. And people are falsifying symptoms to so that they can actually buy probably fairly high quality dope. And as far as I understand in the US its not a manufactured or not tableted version over there it’s mostly bags of grass that they smoke. |  | I rate pharmacists so like in particularly the young pharmacists coming through I’d 22 interns in the last 20 years and they have been fantastic. So I think interns are more capable and they’ve ever been now. And I think I was handing them responsibilities these are really good thing to do, |
|  | I don’t remember being trained on this when I was at uni |  | I'm unafraid of giving pharmacists responsibilities because I think they are better than they have ever been now. |
|  | there’s the issue of stigma, there's the issue of having adequately trained staff, there's the issue of making sure that all the protocols for dispensing it are followed and maintained. |  | If it does become legalized and we do use it for it's medicinal properties the stigma around it would kind of be uplifted, it would be a lot lighter. I guess when you say marijuana people just think of you know people holding a little stick and smoking it. |
| Lack of awareness | as the first point call needs to be educated appropriately. They need to know about the medicinal cannabis itself. How, you know, it's half-life? How much of a chance of addition, of abuse? What doctors are involved, like I was mentioning earlier? Once all of these are established to the pharmacy community I think they can stand sort of firmly all together and sort of have a uniform view on it all. |  | Its just the more normalised it is for cannabis in regards to its moderate beneficial use be it medicinally or recreationally the less of a stigma it will carry. |
| Lack of awareness | Uhm no, but I'll just ask you a question. Is there like a, is there a timeline for them to try and legalize this? Is there any new about this? |  | Yeah, I guess having it like prescribed or something like that from doctors and having it administered in the pharmacy that would definitely decrease the stigma. I guess that adds a bit of creditability to it instead of having like in the US where they have marijuana clinics where it's just people who come in, they buy their pot or whatever. If it comes in like a pharmacy then it might change people's views of it. |
| Lack of awareness | We haven't really gone into the cannabinoid receptors. I think can you use this for Alzheimer's? |  | to be honest I haven't heard too many people uhm express negative view about it. We've actually had some patients ask about do you know where it's at this stage because you know they might have a child. There is one child we know, we won't name the name or anything, but they have some kind of neurological condition and the professor has actually mentioned something about cannabis and uhm hopefully that will be a future treatment for their condition. |
|  | I'd like to know, is it addictive? Cause I've heard a lot of things that it's not really addictive compared to many substances that we use like nicotine. |  | I don't think there's that much negative stigma. You know, even if there is uhm people will get over it. You know what I mean? Once they see it's actually benefits in their community it might pay their perspective. |
|  | I think its crucial that all members of the health profession are able to answer any question on cannabis and provide evidence based responses. |  | I don't feel there is alot of stigma to be honest. I think there's more like a push for it to be available. I've seen a lot of stories where it's been beneficial. So maybe with my health background it's like I don't see it that way. Like I see it as helpful but maybe just who's without a health background they might have a different view I guess. |
|  | I'm a pharmacist I don't know much about it. So if you're asking a normal patient I don't think that they even know that it could even be used for MS like you said. |  | if you do it in community settings you will take away a lot of the stigma associated with it as well, because we have seen with happens with just umm the OSP in clinics and hospitals they are not best environment to go to and that’s the same thing for a person who palliative or in pain. |
|  | I would love to research into and to see if there's any clinical trials or studies to see whether or not it's actually effective for pain or whatever indications it has. |  | I think it’s slowly, slowly going away as a stigma I think it’s becoming more accepted as part of normal culture, |
|  | the prescribers also need to have the appropriate training as well... because you know with opiates you often find the prescribers that are inappropriately prescribing and are prescribing under duress as well, so I think there needs to be that training or additional training to help supports them with that because that will definitely... it will sort of make or break the program |  | Well I'd say it's more just as learning about new medicines. That's all it is. There's always new medicines on the market and so if they would produce something for marijuana in the market then just go learn about it and make sure you know about it and teach people about it. |
|  | - So it's hard for me to say yes it should be legalized or no it shouldn't. I don't know about it's legal status. - I think you can use it in a hospital under restricted circumstances. - I don't know really much about it, to be honest with you, because I haven't had any experience with it. I don't work in a hospital so I don't know how they obtain the cannabis and I don't know, obviously, if it's effective - I don't know uhm cause I'm not exposed to it that much - Are they for or against? - if you've got any evidence behind it's you know, safety, efficacy or that I'd love to have a read of it. |  | I think we (pahrmacists) are well equipped, I think it’s just general communication and maybe the drug reps. that go to the pharmacies and just explain that it’s coming out on the market how it’s used I think that would be sufficient, but I don’t think we need any extensive training for it. |
|  | - is it linked to dementia? - I am not sure, is it used for pain? - So you know at the moment I see more going to nausea and chemo band palliative care, I didn’t consider it being used for pain, it didn't really cross my mind. |  | yeah like with any other new drug, lots of things you need to go to a lecture or study on line or read it in a book, it can’t be that hard surely. |
|  | - I don't know a great deal about the pharmacokinetics of it, I believe there is merit to it, I believe there could be a lot of benefit to it being legalised. - Because as far as I am aware there isn’t enough evidence based information readily available for us to make an educated decision, as far as I am aware it may be out there but I am not aware of it. - Interviewee: Do you know how far away clinical trials are? - Interviewee: Is there any evidence from around the world that has been collated? - Interviewee: So it’s currently being used in some Countries for those purposes? - Interviewee: So there must be a fair bit of data to support that that has already been collected? - Interviewee: So it is legalised for that purpose already? Interviewer: Yes it’s registered under the TGA by Nevadis Pharmaceuticals. Interviewee: And how do they distribute it? - Interviewee: Okay right so it is actually available in Australia in a spray form. Interviewer: Exactly yes. Interviewee: Packaged, ready to go. Interviewer: Ready to go just waiting for that legalisation that’s all, so it’s really interesting isn’t it? |  | Pharmacists do need to be trained how to legally responsibly, ethically supply medicinal cannabis to patients, so there needs to be training involved, there needs to be guidelines set for pharmacists |
|  | I’m absolutely open to it, the fact if someone can show me where it fits in. But I’m unaware of it being of it being necessary to me it feels a little bit trendy rather than a filling a valid hole in our array of medication. |  | We definitely need to be trained like more, yeah its something that I think needs time for everybody understand fully. |
|  | Obviously with pharmacists they want to CPD points whether it be at the APP conference or whatever it may be |  | It’s time for a shift in the way we think about it |
|  | It's going to be in a highly regulated medication. It's going to be appropriately. It's going to be monitored and tracked. Then I think people would have a different feeling, you know like wash out that stigma attached to it. |  |  |
|  | Educating people about it uhm. It may be a stigma at first but I think with further education and think that would change people's perception of it. It might not be the best word to use if they are going to make medications and call it cannabis. Maybe another term or something.  Yeah and you know how it can be detrimental to some people and things like that, because the idea of medicinal cannabis has only come out recently, I don’t think a lot of people are aware of that and they are not too informed of it, |  |  |
|  | If you had any sort of medical training your perception of going faster and going slow Central Nervous depressions and Central Nervous Stimulus, and you can see that they all fit in the same thing. Like people, the general public are generally really, really shocked. When they find out that codeine, oxycodone are opiates like heroin that gives in a real... massive shock |  |  |

| **QUOTES Cont.** | | | |
| --- | --- | --- | --- |
| **Comment** | **Barriers (-)** | **Comment** | **Facilitators (+)** |
|  | It depends on the patients that are coming for it [cannabis]. We don’t do methadone here so and say if cannabis follows the same S8 regulation as methadone… well we don’t supply methadone here due to a number of reasons. First we have never really tapped into it, we don’t think that our clientele would be best for this area. We know Liverpool already has quite a few clinics so we don’t know whether it is worth it, wether the remuneration is worth the pharmacist’s time. So as long that those factors are taken care of then we would look into supplying it but it’s a matter of weighing up the pros and cons of its supply. In saying that I don’t want my patient to think that we are a ‘cannabis’ pharmacy it might give us (franchise) a bad look to the community. That all comes back to stigma and if that is all changed then that can be something that can be looked at but there are a lot of “ifs” before I can answer that. I would like to but especially with something like cannabis I would need to have all these answered before I provide an answer as to whether or not we would supply it. |  | - I think if your going to distribute these types of medicines for these particular conditions that we spoke about I think the public would expect them to be accessible and expect them in community pharmacies where they go for other treatments as well so due to that accessibility and the focus being on the consumer and all of those things that you talk about I would think it becomes legalised and ticks the boxes to a lot of those things and I would think it should fit into mainstream pharmacy  **-** that’s why I was leaning to making it as accessible as possible and therefore it should be dispensed as a normal item that you would dispense in a community pharmacy. |
|  | There are definitely harms associated with its long-term use. There is no doubt about that. But throwing the blanket over the whole thing and saying no we can’t use it because of that is kind of a way out and unethical. |  | **-** a trend of changing drug laws in general in terms of moving away from policing, as its not a justice, but now a health issue and it might be the first step in changing how certain drugs are managed and recognizing that individuals should have access to things that would help them with their conditions but at the same time not allowing people destroy their lives. |
|  | I think until we are made aware of the clear hard-core evidence it is very hard for us as pharmacists to be informing patients at this early stage. You can’t recommend to a patient to go and get hemp oil you just can’t because it makes you look unprofessional and says a lot about your ethics. Yes sure we may know a lot about the research but the community doesn’t yet and you can just be like Oh go use cannabis you can’t say that until the evidence is made more aware. |  | with everything to be honest there are pros and cons. The pros, in this case would out way the cons if the pharmacists or the medicinal board that approves the prescribing of such medications or cannabis adheres to the structure and guidelines then I would see no problem. But obviously there are always cracks and faults and some people may abuse the system; specifically maybe the doctors themselves, pharmacists in dispensing the cannabis to particular patients, off label use of the cannabis, outside sales of the cannabis especially because of the purity or strength that would need to be passed through and approved by the government. So yeh of course there would be faults and cracks but I think if they do stick to the majority of the laws and guidelines. I think it’s a good think. |
|  | I tend to think that most consumers aren’t that focused on the quality of evidence that we have through medicinal medicines and in particular a medicine that has got this kind of association with pleasure seeking/euphoria I think people are much more focused on what they hear as anecdotal evidence through social media and print and paper and all other media forms. |  | So I guess it comes to the ways people go about getting access to it. So I think it also has to do with the indication that the patients are prescribed it and the ways regulations… and things are coming about in pharmacy practice to prevent dodgy people coming in for it and abusing it. But it would be different when it comes to medicinal marijuana I guess if it ever did come into practice it would be just the compound that would be available not like… you wouldn’t be able to get “weed” in a bag or something to smoke. I think it is very clear how pharmacy would go about it. |
|  | Where I draw the line is as a pharmacist I suppose is that I don’t believe that the raw cannabis is the right product to be promoted, researched and promoted to be used in any situation, but that it could be that the medicinal cannabis could use extracts of it and be ingested in a way which minimises the irritation for lungs and stuff like that. |  | The reduction in crime associated with cannabis if it were to be legalised and also there will be health outcomes whether they be actual or perceived as I said before for people with pain or cancer. It would also help benefit … by taking away profits away from criminal activity and socio dependent. |
|  | well thinking about recreational use of cannabis so it’s something that I am not sure that the pharmacy profession has a real role in entering, I think that’s a decision between the consumers themselves the general population and law makers regarding it and those decisions need to be made with regard to safety and long term harm and all the other things that go with it, when you think about using medicinal cannabis I think that we do need to be able to look at evidence based approach to do it, I am guessing that the level of evidence of it isn’t very strong and therefore there needs to be some research and if the research comes through |  | - I think clearly when you look at overseas and some areas where medicinal cannabis does appear to give patients benefit perhaps beyond conventional therapy.  - there is some evidence, that is true but I not confident in how rigorous that particular evidence is. I guess without knowing that I would say that the studies in the Australian setting apply the appropriate rigor around a whole range of things from research to formulation to safety etc. and I think if it is time that we go on a new path of legalising cannabis potentially I think we really warrants the undertaking of all that to support for what you are doing in your country. I think we can certainly take on some of that advice and evidence that there is overseas and take that into account but I definitely think we need to do our own work in that particular area |
|  | My understanding in America they can still smoke marijuana and it is legalised. That’s outright stupid. It can be an inhalation if they need it to be fast acting but it shouldn’t be smoked because that is recreation and people have greater risk of abuse and lung damage. So if it is done in a formulation … I’m not to sure if it’s sublingual or buccal? Or maybe even an inhalation something similar to Spiriva maybe. But I don’t know if that can be done. As long as you can reduce the risk of people abusing it I think it is something we should look at. |  | Well, I know in America in some states they have legalised. But the way they have legalised it I don’t think they have done it right. It is still been abused quite a bit from my understanding. I might sound naïve but from my understanding it isn’t well executed and not regulated correctly. It can be done here in Australia, I have read case studies where it has helped people with really severe seizures and has helped with them. So that’s the type of therapeutic outcome you can get and if there is a substance out there - doesn’t matter what it is – but if it can give someone a better quality of life then it is something we should look at but we need to learn from what has happened in America I don’t think it has been done properly. |
|  | Definitely for the majority of the population, there’s a negative stigma to it, it’s as soon as you seen the word cannabis first thing you think of is smoking and everything you see on TV and all the reports have obviously been bad about it, there have been some odd cases where I have heard from overseas, some cases here in Australia too, I think where they have actually prescribed it for patients who are terminally ill or kids who suffer from a lot of medical conditions, as in no other treatment except for cannabis, I mean those particular circumstances I think are forgivable |  | there are plenty of examples of successful models in terms of controlling supply chains, how its supplied dispensed and make sure the quality is there, whilst still managing its overall access. The international frameworks are there and I just wouldn’t be that difficult to implement. It seems like it would be a benefit to everyone involved the main challenge is just overcoming the stigma that has been built up over the past 60 or so years. |
|  | I think just historically because the use of cannabis people associated with being illegal and harm coming from it and all of that type of tape. But I think if it’s going to become a legal part of what we do, education campaigns for consumers for health professional for a whole range of reasons is going to be important. |  | Unfortunately we don’t tend to use trails that have been completed overseas although maybe we should be able to. But I think there is a groundswell within the community because there are a larger number of people now knowing what some of its advantages are. |
|  | of course, there is a certain amount of stigma associated with the use of medicinal… or cannabis. It’s just due to the Hollywood factor and as well all the associated press with cannabis. I don’t blame the general public but I think if they are more informed by the Australian government through ads, pamphlets, more training for doctors and the general health professionals in order to dispel some of the myths I think the perception can change. |  | How funny… I had actually written the PSA policy on medicinal cannabis I think the industry likes to holds itself up as a strong standards based type of thing so I think in that sense the evidence is really important and we should be calling on the gathering of evidence, not to support its use because it’s pretty obvious that its beneficial in various ways, but more so to unify the evidence. And it doesn’t need to be necessarily all RCTs, but in a more organic sense patient’s experiences and qualitative results. |
|  | Some negatives I suppose is that there is still a lot of stigma associated with cannabis particularly with older generations. Just like there is stigma associated with methadone clinics for example, I can fully envisage that initially at least there will be some negative connectivity associated with clinics that deal with cannabis. But gradually, over time I believe that stigma will wear away and there would be growing acceptance. But it would be brave for someone to step up initially and take up that role. |  | trails for cannabidiol and also THC I understand there are trials for nausea and vomiting for cancer patients but also that there well could be significant benefit to some drug resistant epilepsy in children and that perhaps is a little bit lagging behind in research compared with the cancer one but I can see - from what I have read about it that it could be a really positive advancement in its use. |
|  | It’s a combination of both (evidence and media/hearsay) I guess, Its about your upbringing, so if you're taught and brought up... like how it was perceived back in your home country (culture). Obviously the media has a lot to do with influencing peoples minds but I do believe the stigma is always associated with the fact that people are quite passionate from both groups of the ‘yay’ and the ‘nay’ sayers. So I think its more of a moral issue rather than anything else. |  | I think medicinal cannabis certainly has its role… I’m not a user of cannabis but I have heard that cannabis can be useful in certain cancers and that it may be useful for certain neurological conditions such as Alzheimer’s. But those studies haven’t definitively proven its benefit. |
|  | Yes without a doubt. It’s just because it at the moment is only used mainly as a recreational drug. It is considered a gateway drug to stronger drugs of addiction as well. Some people see cannabis as something to do for fun on the weekend others don’t do it because it may lead to other drugs. I don’t think a lot of people know the medicinal potential of cannabis, maybe healthcare professionals do but the general public don’t and you hear stories in the media of what they are trying to portray it with some pushing for it to be legalised others in the past highlighting it negative effects and this influences a lot of people. Even though it might not be the best information source it can still change the way they think. |  | Often it’s the community saying that “we want this” that urges it along but I don’t really know what its like right across the profession I really can’t say that and I gather that the medical profession has a few reservations and really wants the trails to be completed before its legalised. |
|  | In saying that I don’t want my patient to think that we are a ‘cannabis’ pharmacy it might give us (franchise) a bad look to the community. |  | I do see a massive difference between the two. So if it is medicinal it would have to be under strict laws, regulations and guidelines it would have to adhere to that. Obviously for recreational use you would know straight away with either… not only teenagers but also with adults for their own leisurely use and that is the large difference between the two. |
|  | I don’t know I think that it’s fading. I think the big epidemic with ice and that is taking over. People don’t really perceive marijuana and a harmful drug anymore. If you look at statistics not a lot of people die from marijuana use. I know that overtime if you smoke and use marijuana illegally in the recreational sense everyday for a significantly long period of time then obviously that can change the chemical structure and functioning of the brain. But use medicinally I think would be very very different. And I think like with the problem with ice I think maybe we should move that perception over there (towards ice) and not stop something like medicinal marijuana from helping cancer patients. |  | the distinction between medicinal and recreational cannabis is that one is being controlled by a healthcare professional and the other is self determined so I think there is a role for the clear cut distinction. |
|  | Yeh absolutely, I think there is stigma associated with it, I mean there wouldn’t be many people I’d say that have a good recollection of cannabinoid products being available. It’s very similar to that whole debate that vaccinations cause … bad things. A lot of that is because there is this lower perception of risk of vaccine preventable disease because people haven’t actually experienced it. And I think there is a general mistrust in the community that I think – this might just be an example of how well western manufactured medicines have been explained to the community – but I feel there is a general mistrust or apprehension about using something that is grown. There is a disconnect between where things come from and how they end up in something that people consume. |  | First of all it should have a therapeutic outcome and should be used for this health outcome and not its euphoric feeling. My understanding is that cannabis has a lower risk of addiction and harm, with people simply seeking its euphoric effects but the distinction is when you’re not worried about the therapeutic effect but more concerned about its euphoric effects. I think that is where the line is drawn. I think its similar to our oxycontin, we know it’s a great drug to be used for pain but its when people aren’t using it for pain relief but rather to maintain a normal life I think that’s when the lien should be drawn. Having said that as long as they are using it for its intended purpose as long as it is not being used outside its prescribed indication. I think that where we can also draw another line. |
|  | You will certainly get people who are shocked by it or whatever, where they have had little exposure to people using it or some don’t realise that there are people around who are using it. I think there will always be that to some degree but overtime that will all change I would expect. |  | I would think it is probably a schedule 8. Although I have seen some recommendations where depending on the type of cannabis extract you use could perhaps have it as a schedule 4. But I’m not an expert in the area. Where perhaps S4 or S8 well that would be determined by the trials, the quality of the products and coordination. Like I’m flexible in that area but it would probably enter as an S8 or S4. |
|  | It just that I do think that your standard community pharmacists wouldn’t have the adequate training after finishing uni and registration to just go out and dispense cannabis. But definitely some additional training is needed |  | I would be happy for it to be legalised so long as it is properly regulated and controlled I guess in its quality and also the amount that is actually to be consumed.  Reference 2 - 1.38% Coverage  I think there would be less crime if it were to be decriminalised and legalised to be honest. |
|  | I don’t think a lot of people know the medicinal potential of cannabis, maybe healthcare professionals do but the general public don’t and you hear stories in the media of what they are trying to portray it with some pushing for it to be legalised others in the past highlighting it negative effects and this influences a lot of people. Even though it might not be the best information source it can still change the way they think. |  | I think if it follows the same guidelines as the dispensing of methadone or suboxone and a pharmacy would have to train pharmacists in a particular program and get registered in that program with the particular software as well along with that I think that would be the best way forward in that. Obviously it may need to be more highly regulated and more check-ups would be needed by an overseeing body in the actual dispensing of the medication and things like that. Yeh I think if they follow the same route as the methadone and suboxone or buprenorphine program sorry… it should be completely fine. |
|  | I think it’s a little bit of both truthfully. Media gets little snippets here and there from small studies and little evidence and make it kind of a big thing. Oh marijuana does this… marijuana does that but no its not. They know very little about the bigger picture, the studies going on in universities finding big, big improvements in people’s health outcomes that they are not aware about. |  | I think it would be in a schedule 8 setting… if you change the system or rock the system too much then not many people would like it. As long as you keep it within the system then I think it would run. |
|  | I guess all medicines have that balance between risk and benefits and it’s up to us to educate and inform patients about that balance which is no different in cannabis. We also need to remove the debate from the perception that the only people investigating and advocating its use are just a whole bunch of hippies because it’s just not true. Its just a lot of community re education that needs to happen and that’s not a short process. |  | Once or if it does come into community pharmacy it should be a DD, S8 medication if not higher. So they might need to bring another schedule in and make it even tighter… I don’t know how much tighter they can make it. I’m thinking direct contact with the doctor every time something has been prescribed, no repeats at all just constant follow-ups with each healthcare professional to make sure it’s not being abused. |
|  |  |  | As long as there are regulations and specific, certain doctors being able to prescribe it just like things that we do in practice for other medicines like Ritalin and Concerta and medicines like that. We check the doctor their reference numbers in terms of being able to prescribe certain medicines and from there we dispense it. So I just don’t see why they can just do the same thing for cannabis.  I think that once the pharmaceutical companies get their research together and it has passed all the clinical trials and there is … you know… really good clinical based research showing its clear benefits and goes through all the proper manufacturing requirements umm so once that all happens and the TGA has obviously approved it then I guess regulations will come into place for pharmacists to dispense it as well as deal with how we are to dispense it in terms of protocol and how we are to manage it and its pitfalls with abuse. I guess it all has to do with regulations. |
|  |  |  | Yep, so currently in Australia it can be used with approval of a minster and that’s about it. Schedule 9 I believe they call it so there would have to be… there is a lot of red tape around it.  or instance if we were in a situation, either federally or even state level where the possession and use of marijuana was decriminalised or legalised so that people could purchase it that wouldn’t be something I would see in a pharmacy. It’s the same way that cigarettes aren’t in pharmacies. That would be something that people consider specific, like they have in America they have a cannabis dispensaries as opposed to being in a pharmacy. However therapeutic products those ones should be dispensed and have all the same requirement that schedule 8 products currently do now.  do the legal framework to make it a schedule 8 product so look at where the use of it is going to go but also they would need to balance that against the actual affect of the medication. So if we are talking about a medicine that can’t actually be diverted and can’t be abused in any method then it should be a schedule 4 product instead of schedule 8. And if it is a product that has the ability to be abused so it has a high level of delta 9 tetrahydrocannabinol (THC) in it then it should be a schedule 8 but they should do all the legal frameworks once they start looking at having the products available for regular use then they should schedule it into 8 and do what they can to make it legal. |
|  |  |  | I’m very happy for it to be legalised but I see that there is an importance in having it standardised before it is legalised. You know you can’t just grow a plant and then just use it because obviously there are variations in the plant itself and in the growing conditions so I think it is very, very important that it is standardised. I see it as being a medicinally use product… I guess much along the lines that digitalis was standardised and used. I mean over the years pharmacology has developed form plants and its a lot better when we have standardised and know what the ingredient is. And there is a whole range of ingredients in cannabis so it does need to be standardised.  I would think initially it would probably need to go into schedule 8 umm it does make it more difficult for access but umm I suppose I have seen abuse of … even things in S4D. they are harder to control than schedule 8s and pharmacists are quite capable of managing schedule 8s so I would thing that would be a great way for it to go. However given that I would want it to be nationalised and that isn’t the case with all medications and I would think if its was going to be scheduled then the same requirements would need to be met across the country.  my main concern was I guess from a legislative point of view is that when it comes in it is standardised right across the country and it seems as though some states are hurrying this along more quickly than others and that in itself is an issue in the long term. So if we have got young pharmacists understanding its use then I think they can help to move it along. Unfortunately the trail they will take a while and I don’t think the legalisation will occur up until the trials are finalised. |
|  |  |  | it has already been practised in, I believe, 4 states of Northern America. So Alaska, Colorado, Oregano and Washington I believe have already legalised it and they have a massive demand for medicinal cannabis there. So public opinion does sway from time to time but I do think eventually they (public) will take that into consideration and have a good hard think about legalising medicinal cannabis. |
|  |  |  | Like in the odd occasion you get the terminally ill patient with cancer and they come in and ask for empty capsules and after asking what they are using it for and they say for hemp oil or something like that. And they see such great results from it so I think that even though it is illegal, marijuana is illegal, the cannabinoid components of it they are medicinal. It is working for people in the US why not here? Why should terminally ill patients have to suffer when there are things out there that can really help the? |
|  |  |  | And I guess always having the consumer at the forefront of what we do there is benefits for consumers in medicinal cannabis where if they don’t gain it by what we consider to be conventional therapy you would certainly investigate it. That is why I am support of probably trails that are now being suggested uhh… and I think now in NSW it has been put forward there… to investigate the use of medicinal cannabis. |
|  |  |  | I think there is a place for it in the health sector. I know there is a big stigma towards it. There have been cases where cannabis has helped out and have given good health outcomes for patients, which is what we are ideally here for. It doesn’t matter the way its grown or abused, if it gives a good health outcome with minimal side effects then I think there is a good position for it in the health sector. |
|  |  |  | I think that… it’s got a lot of promise and its only really because of the increased use of if recreationally that the medicinal use of it has fallen by the way side. I think that it has been demonised to a certain extent and it should really be treated in a different way to any other drug that is used for therapeutic means. Yeh I don’t want to go on too much but I think it has its place there should be more work done on it and trying to get or gather the right sought of evidence to support it. |
|  |  |  | My general thoughts are that any therapeutic product that can be looked into should be looked into. So if there is a potential for cannabis to provide therapeutic benefit then it should absolutely be looked into and later implemented.  The main thing I’d say is that if it fails to get up because of public fear that would be a huge lost for a number of people who require a product that would change their lives. That’s the main thing we have to look at and its not going to do any harm to anybody… from my understanding these products are not that divertible or desirable to people who want to divert it any way due to its formulation so the hype of not using it is unjustified |
|  |  |  | I think pharmacists would be well equipped and trained enough to deal with any formulation…maybe the only one [exception] would be injections would require much more training on the pharmacists end and I know that we are now doing vaccination trainings and we are able to do vaccinations if you are properly registered and trained in that regards but about injections on sight I think that would be a bit too far fetched and maybe only if we are extensively trained and the boards approve of it then I see no problem. |
|  |  |  | But I assume it’s also the amount and… how we should go about policing. I guess if you had more studies to look into what amount of cannabis is medicinal then we would be able to set a more clear cut therapeutic amount as to how much becomes medicinal and how much of a certain amount be recreational. |
|  |  |  | The community has moved away from that, and there are benefits to that, but I think certain things that would be useful to communities fall to the way side and I feel cannabis is one of them. And based on what I know how cannabis works as a plant, it really needs to remain in its full form to work and it just doesn’t work if you take out certain things in isolation. So I think the investigation is more going to be, along the route of what has been done in the US and Netherlands which study compounds and know, definitely, what concentrations of each compound are in it, but it would remain as a botanical product. |
|  |  |  | I definitely think that a pharmacist should have a say in this, because obviously we are all part of the health care professional team and in order for us to actually help the patient we need to actually work hand in hand together and have all different types of opinions kind of amalgamated into this, yeah because at the end of the day everyone just thinks that we just dispense medications.  We do have a medical background as well and we studied this for many years as well, so we are not just behind the computer typing the labels you know and issuing up the medications, so I definitely think we should have a say in this particular, in everything actually, I mean. |
|  |  |  | I would think so, because I think, again we always say that community pharmacy is one of the reasons why we rate highly and play such an important role in the community which I think is acceptable  I think it is a key role for the pharmacist to do and I think due to the reception and sensitivity of the word cannabis. I think there would need to be a specific campaign around why you can now get cannabis for certain conditions if it turns out that way from you local pharmacists with a prescription from your doctor.  Umm that’s an interesting question… I guess it’s a bit like, probably methadone. We (PSA) encourage the profession to dispense methadone but not everybody does it and some people go into it in a much bigger way and it becomes a business part of what they do in their pharmacy with probably large numbers involved in the program. You cant tell everybody to dispense cannabis. If for certain reasons they don’t want to dispense it. It could be safety as one reason (as we spoke about) it could be they don’t want to place their staff at risk if they carry and dispense cannabis within their pharmacy. So I don’t think we can dictate that all pharmacists dispense it cause individuals may have their preferences and that is something we may have to accept. And I would accept that. I think we should encourage all pharmacists to be part of it and participate in it... and as we go back to the bit about consumers, acceptability by consumers and pharmacists appoint their health needs. That sort of campaign should say that you have a responsibility here to dispense these particular products but at the end of the day I think the person will make their own decision on that. |
|  |  |  | I do think we are generally conservative in lots of debates probably and I think that shows a little bit by historically when we talk about the health industry and we talk about doctors and allied health often committees get set up, whatever and often pharmacist have been missing. And I look back at years where health committees have no pharmacists on them and they have been missing of the list and the reason for that, I think its the environment we work in, more of a retail environment where pharmacists are business people as well as health professionals. And when you think of allied health you tend to think of physiotherapists and podiatrists and you think of mainstream medical profession you think of nurses and GP that’s what they do all the time as their full time job. Pharmacists sort of have that mix of what they do and we sort of have that umm… get left in our own little world a bit sometimes and people are not aware of the skills that pharmacists have even the discussing this just in the forum that I was at often there is a lack of understanding at what pharmacists actually do in contribution to the health system. And I think if we go back to the pharmacist again we are not proactive in the community and other health professionals in the skills we have and what we can provide and I think that’s why we are generally a conservative professional… yeh.  I think it needs to come form the peak organisations… I think these days with social media and what have you. In my serve as PSA president, even when I started to when I finished I think pharmacists where engaged in a lot more through social media then when I first started so I think there is a capacity there for that debate to happen and I think it’s probably best driven, initially, by the peak bodies. So the PSA, Guild, SHPA, PPA… I think we all have a responsibility to drive the debate. |
|  |  |  | As a pharmacist I wouldn’t mind to be honest, the more roles or the more opportunities that the government, I guess approves first and then hands down to the pharmacists I would carry on… carry the torch with it to be honest. If there is benefit that is greater than the risk then I’d be more than happy to carry the torch in this case.  Reference 2 - 10.83% Coverage  As a pharmacist to be honest we need to know our boundaries. In this issue our boundaries would be in the administration and handling of the cannabis. I don’t see it as our role to promote the medicinal/therapeutic use of it that would be up to the doctors or the medical professionals in that area to voice their opinions and clinical guidelines in that respect. I don’t see us voicing our opinion too much about it besides I guess the humane factor as ever person can of giving people equal opportunities or equal rights in regards to the use of cannabis or not. But in regards to pharmacists specifically personally myself I would stick to more of the administration and handling of the actual cannabis itself that’s where I would voice my opinion.  I guess we should embrace any changes that come our way. I guess in the community setting because it’s more … business oriented not so much patient oriented as much as it use to be back in the day. So I think anything that can enhance our role and benefit the patient for them then we should take it on board. |
|  |  |  | I think the pharmacist has a pivotal role to play in this. I mean ultimately we are going to be one of the first health care professionals dealing with this second to only the medical practitioner who prescribes it. Look… I think its something we should have a decent debate over, certainly not a topic to be taken lightly but I think with the proper training, community pharmacists can definitely have an important role to play in the legalisation and medicinal use of cannabis. |
|  |  |  | I think pharmacists are being undervalued in the community ad there is a hell of a lot more that we can do then what we are currently doing so we don’t need to be more equipped per se it is just that we need to initially understand the scheduling and everything about the medication itself which is the same for any drug like a new diabetic medication. We do need to understand all that but I think we are already well equipped otherwise and we are not doing as much as what we can and should be doing for the community.  Our voice as pharmacists it should be heard because we are the ones dispensing it just like with, methadone. I don’t supply it but I have pharmacy friends and family who do supply methadone in their pharmacies and I do here stories of people getting angry… whether it be doctor has written the script correctly… what ever it could be … we are the ones that are getting all the anger from the patient at the frontline. So if cannabis is to be legalised we will be experiencing the same thing so definitely we should have our voices heard if we should do it or not. Having said that if we have enough trust in our bodies of pharmacy, the PSA, the Guild, whoever is getting involved in this, if they give us the right scheduling and the right platform for it then it can be done, but I think it needs to be talked about a little bit more and needs to be actually… umm I don’t know not a vote... but I think our voice does need to be heard but if it is to be implemented all the platforms and all that needs to be right to help the pharmacists. |
|  |  |  | We should be pushing to be involved in it I think. We should be looking to be engaged and part of the whole process from the very, very beginning, on that note we should be trying to pose solutions in terms of training and development so we can be proactive rather than just backing away and saying that its just too hard.  The pharmacy industry is very conservative. It hasn’t had to be particularly innovative or anything for a long time. People don’t like to change, I suppose. So this is a key opportunity to be ahead of the ball game and contribute. I think that PSA in particular have a big role to play and the universities as well in getting this sought of stuff [cannabis] back into the curriculum. It such a cheap substance and the trends of things is that they are getting astronomically more expensive and cannabis is cheap and safe, so it should be investigated to its fullest potential. Pharmacy has always been about being an accessible and advice driven industry and it has always been about patient advocacy… So I think in that sense we should be taking lead on that and working with other groups to try and it out and rolling. |
|  |  |  | I think that pharmacists voices are important because pahrmacists have a specific ability to cut through the hype… I guess… one way or another. And look at it from a therapeutic point of view and again a similar reflection of the same issue would be the use of amphetamine on prescription and the current media coverage of its adverse effects and there are a lot of views on that yet we are not talking about removing amphetamines of prescription.  that is always going to be the case because… umm… doctors are perceived as problem solvers while pharmacists – and this is from our end – as just performing a role hat people take for granted so therefore… Like if you were to ask somebody who would you ask about medicines for advice, they would immediately say “oh I would go to a doctor” and then if you asked if they would speak to a pharmacist then they would say “oh yeh actually I would.” So its like second nature and taken for granted we are so integral to peoples lives that they just don’t think about it. It’s sort of a separate issue but I’m always talking to that PSA about having a visible person to be the voice of pharmacy just the AMA quite often does. There are a lot of celebrity doctors for instance. But uhh… its not so simple you have to find someone who is good at being a pharmacist who is interested in being visible and that is the opposite to what most pharmacists are. We are wired not to be quite upfront and are comfortable in our roles as quiet helpers of the healthcare system. |
|  |  |  | Well I would respond by saying that I was very conservative, however I am an older pharmacists and until I had attend PSA15 Expo I was extremely conservative however pharmacists do respond very well to continued education and being informed about this I would think as soon as pharmacists become aware of the advantages of medicinal cannabis they’ll take it very well. And younger pharmacists are a lot less conservative than the older ones and even older ones can change. I’m an example of that. [Laughs]  Well ideally, sorry I’d have to correct you there, the profession not the industry. [crosstalk/laughs] certainly it would be ideal if the profession held a united view there is a body in Canberra which has representatives from a whole range of pharmacy organisations; Guild, PSA, SHPA all of the bodies. Now if that could come out with a united statement that would be ideal. But I think the likelihood of that happening is really quite high because I think there has been an international push for the legalisation of medicinal cannabis and we are learning from that so I would think there would be quite a willingness to accept that.  I think it depends on the age of the people that you are talking to and their education level. I mean a few years ago within pharmacy I’ve had people with teenage children – in their early 20’s – that had developed schizophrenia. And there is a feeling within the community that people can develop schizophrenia from the use of cannabis. That’s now seems to be refuted in some fields, but as long as people have that opinion then they are going to be quite resistant to it. But I guess not many people would express their use of cannabis, as it is illegal at the moment so [crosstalk]. Now I certainly hope that pharmacists will be the suppliers of cannabis and I feel that they are well equipped for the job. |
|  |  |  | Say if pharmacists do dispense cannabis legally for some of those conditions we spoke about. I think that’s were there will need to be later an education campaign for pharmacists, the consumers and probably all healthcare professionals around this issue of cannabis is legalised for these sorts of conditions. We don’t support the illegal abuse of cannabis and we need to make that clear.  the education, information and public campaign it needs all of that and I think there is a raft of things that goes with informing people appropriately if we get to the stage of dispensing cannabis as to what the really means. |
|  |  |  | I guess so but that’s just like with any other drug that comes into the market. We are informed more about the drug through bodies and the pharmaceutical company’s reps that come out and all the pamphlets with all the pros and cons and side effects, contraindications and all that sort of stuff. So in terms of training if it goes down like how all other drugs enter the market then there shouldn’t be a problem.  Yes, definitely. Well I mean I saw how easy it was to come to a greater understanding of medicinal cannabis from the PSA Expo and younger pharmacists that are around have a far greater capacity of comprehending this and the pharmacology and to educate the public so yes I think pharmacists have an ability to learn and then disseminate the information. |
|  |  |  |  |
|  |  |  |  |

| **Setting** | **QUOTES** |
| --- | --- |
| Specialised Cannabis Suppliers (SCS) | I guess definetly having it in hospital or community or maybe having separate dispensaries similar to what they had in the US. I'm not entirely sure on what the right answer is. It's something that like I'd be happy to discuss and my view as a pharmacist is that I'm happy to be swayed by the best possible evidence. |
| SCS | kept in a clinic specific for just cannabis or marijuana, probably in a hospital and if there are areas which a lot of people are relying on the medicine for it's medicinal uses then possibly just a clinic just devoted to marijuana, yeah. |
| Clinic | So I guess eventually there will be clinics and you know, I guess pharmacies won't have too much of an involvement in that |
| Clinic | Maybe like in a clinic. I just think it can be monitored and uhm it'll be good. It'd be like someone, going to a physio... someone... you know you could go to that place to get that if you need it. It could be a place that's known for it and available medically there's nothing dodgy about it, its all legit. |
| Community | I mean I just take as if it's going to be like any other S8 drug and it would be best suited in a community pharmacy setting for access reasons. |
| Community | It should be within a community setting. I think that all palliative care, there should be something better in terms of accessibility of palliative care in cancer treatments within the community.  I think you need to take the stigma away from that and I think people need to change their dosages very quickly as well. And you know it's much easier with and accessible within a community setting. You know that might happen on a weekend. That might happen early morning or late and night and the family, in order to go back to the hospital where it's nine to five to pick up their medication makes it very difficult for them. I think that's just an extra layer of stress that's not necessary in these people's lives. |
|  | you know we deal with these substances every day. You know we have opioids. We have methadone. We have, you know, ketamine, you know, we deal with things, illicit substances every day and we do it well you know. And so I think that the place to get it is within community pharmacy. We actually know uhm how to read the prescriptions. We have good relationship with the GP who's prescribing it as well. You know I think as E-health becomes more and more accessible, you know, I think there'll be a way to ensure that the authorities know who's using the substances as well. They can see patient histories associated with it as well. So I think, yeah, there's a chances for it to be abused but I think community pharmacy, you know, knows that best and is ready for it?  All palliative medicine needs to be provided in a community pharmacy setting in order to reduce health system pressures and decrease stigma and link with hospitals in regards to palliation and death. And more importantly to ensure palliative patients get a QOL that is comfortable to them and in their own homes with family. |
| Community | I would, I guess see it a little bit like methadone type of dispensing if you like. That would be in a community pharmacy setting. I think community pharmacies give very good support to those people receiving methadone and similarly with cannabis. I would see it and it should be a standardised benefit available in community pharmacies just like dispensing any other medicine. I don’t think it should be in a clinic or somewhere like that, other than … like I said … if there become a safety issue concern and community pharmacy staff in pharmacies are put at risk then if in some point in time they see it fit in a clinic setting or something where it would be specially set up for that particular purpose. So initially I would see it fitting into mainstream pharmacy but depending on how that stages out perhaps. |
|  | I think if your going to distribute these types of medicines for these particular conditions that we spoke about I think the public would expect them to be accessible and expect them in community pharmacies where they go for other treatments as well so due to that accessibility and the focus being on the consumer and all of those things that you talk about I would think it becomes legalised and ticks the boxes to a lot of those things and I would think it should fit into mainstream pharmacy. That’s why I was leaning to making it as accessible as possible and therefore it should be dispensed as a normal item that you would dispense in a community pharmacy. |
| Community | Yeah, I guess having it like prescribed or something like that from doctors and having it administered in the pharmacy that would definitely decrease the stigma. I guess that adds a bit of creditability to it instead of having like in the US where they have marijuana clinics where it's just people who come in, they buy their pot or whatever. If it comes in like a pharmacy then it might change people's views of it. |
| Community | it should be available if community pharmacy as long as pharmacists are aware of the legalities behind it so perhaps there is a specific training for pharmacists to be able to, for example with our QCPP. Say if there's something integrating with that in terms of the pharmacist's procedures and they follow procedures around in cannabis prescriptions and that it is locked in a safe and it's monitored just as if, like oxycontins are, you know S8s. If you restrict it to hospitals you're just going to be uhm hospitals will also be a place for the people who want to seek the drug illegally and hospital doctors are under a lot of pressure and sometimes they cave in to writing S8s. We know that because we see it in practice as well. So I think leave it in the community. |
| Community | Because it needs to be available to people that are suffering from nausea from cancer and they don't want to have to go to the specialist clinic every how ever often, yeah. |
| Community | if you do it in community settings you will take away a lot of the stigma associated with it as well, because we have seen with happens with just umm the OSP in clinics and hospitals they are not best environment to go to and that’s the same thing for a person who palliative or in pain. |
| Community | I think especially in a community pharmacy because of accessibility so you always want to just make it easier for the patient and I think if it’s just in a hospital or just in a clinic I think that can make it a bit more difficult so having it more accessible is really important. |
| Community | I think it should be done here, that’s my view and it should be done in pharmacy though that’s the way I see it, I don’t see anywhere else it could be done. If in a hospital or something like that, I think you have to be very careful with it. |
| Community | I would say community, at the moment, community pharmacy does like methadone and suboxone, I work in a pharmacy at the moment, I work for at the Alfred hospital, but I also work in a community pharmacy that has over 200 opioid dependent patients and they are on the program and basically if we provide that service in the community why can’t we provide you know medicinal cannabis because they are sort of within the sort of, in my opinion categories because a lot of the patients who do abuse drugs also do abuse cannabis, a lot of my patients do that and they talk to me about it and so I don’t see why medicinal cannabis wouldn’t be in that category, I think it would be communities to be a point of supply |
| Community | I suppose I would see it as being implemented in a system very similar to buprenorphine and methadone, initially and maybe spreading out to all community pharmacies after that. I think a hospital setting would be too limiting for patients to access and same as clinics, I don’t think clinics would be widely spread enough to make them convenient enough for patients. |
| Community | Yes I think community pharmacy is key due to accessibility, I think – I don’t think that’s been said about the 5400 pharmacies that provide the community service obligation (CSO) to the Australian community. I think that’s a network that we should be really, really proud. |
| Community | it should be done with someone who actually, the pharmacist has to probably have an interest and they don’t have to do it in every single pharmacy but the ones who choose to but I can’t think of a better place to do it, any pharmacy really should be able to do it, |
| **Community** | I would, I guess see it a little bit like methadone type of dispensing if you like. That would be in a community pharmacy setting. I think community pharmacies give very good support to those people receiving methadone and similarly with cannabis. I would see it and it should be a standardised benefit available in community pharmacies just like dispensing any other medicine. I don’t think it should be in a clinic or somewhere like that, other than … like I said … if there become a safety issue concern and community pharmacy staff in pharmacies are put at risk then if in some point in time they see it fit in a clinic setting or something where it would be specially set up for that particular purpose. So initially I would see it fitting into mainstream pharmacy but depending on how that stages out perhaps.  I think if your going to distribute these types of medicines for these particular conditions that we spoke about I think the public would expect them to be accessible and expect them in community pharmacies where they go for other treatments as well so due to that accessibility and the focus being on the consumer and all of those things that you talk about I would think it becomes legalised and ticks the boxes to a lot of those things and I would think it should fit into mainstream pharmacy. |
| **Community** | community pharmacy, mainly because everyone should have access to it. It’s not fair to be just like… oh it’s in a hospital but what about the people who live in a regional area, what about those who don’t have access to a hospital. And everyone know what the hospital system is like, its already so congested and takes forever to see a doctor, its pretty bad. |
| **Community** | So I would see it in terms of access community pharmacy would be the best but it is important to get that training and knowledge for the people who are dispensing it and using it and that is the most difficult part of starting something like this at first. The other thing is that people already use it medicinally and trying to integrate those people into the system rather than imposing the system on them is crucial because they are going to use it regardless. So I would see that certain prescribers would have certain training and then they would be able to prescribe it and certain people would be able to dispense based on certain training. |
| **Community** | I think community pharmacies definitely should be one of the places that medicinal cannabis should be at. Patients that will need to use that product would need to be using it chronically and access via hospital arrangements for chronic medications is strort with its own issues like access, especially with country patients. So on a similar note the way schedule (section) 100 products are currently going off hospital supply only into community pharmacies now is a good thing, and I would think that the therapeutic use of cannabis it should be going that way even if it doesn’t start that way. It should eventually get there. |
| **Community** | if I had a child with drug resistant epilepsy the I would want to be able to go to the local pharmacy and local pharmacies are more than capable of handling it. Now whether it can go straight into the local pharmacy or in the hospital to start with I see no reason as to why it can’t go into a local pharmacy because if it’s a standardised product then I would assume it would be made to help reduce the psychological content and have the medicinally necessary content. So I think pharmacies would be in an ideal place to manage it. And I know if I was a patient I would want to go to the local pharmacy.  But I certainly think pharmacy would be able to manage it and it would be a shame if it didn’t go through pharmacy, whether it is community or hospital to manage. But I don’t think it needs to be in clinics. Ideally it would have to be in a community space because a large number of terminally ill patients are in the community and they would want to be able to access their medicines through their community pharmacy and it would be no different from handling narcotics I don’t think. |
| Hospital | maybe just leave it in a hospital pharmacy setting. That way it's more monitored and more controlled. But perhaps if a review is conducted into how they would be able to apply in a community setting then I suppose why not, you know. |
| Hospital | It's not something that, I think, community pharmacy, there is a sort of avenue for that but most patients who are having that palliative care or have conditions like cancer would mostly be in hospital settings anyway |
| Hospital | the majority of it would be in a hospital setting but you also have when the patients get sent home with palliative care and we do get a lot of those scripts at the moment so I do see possibly a small portion in a community setting but the majority in hospitals. |
| Hospital | let the Pharmacy Department in the Hospital that they can dispense it but not like the community pharmacy to dispense it. |
| Multiple | I think it should be available in both community and hospital, because you know accessibility has got to be a big issue, |
| Multiple | I think it would be suitable all 3 settings (community, hospital & clinic), like I said as long as it’s regulated well and you know you have got the pharmacists who are trained well and have enough knowledge around it to provide it, I think it would be suitable in any setting. |
| **Multiple** | I personally would be more comfortable only dispensing it in a hospital or clinic setting, for the reasons I just mentioned, because it’s in a more controlled setting, whereas in the community, as you know opioids at the moment are being heavily abused and I think it will just go down the same path as well if we were to dispense cannabis. |
| Staged implementation | yeah why not be in a community setting but maybe for now, to get used to it all, how people will react to it may need to settle for a hospital setting with doctors around. It just depends on how the trials and how people have dealt with it and things like that. |
| Staged implementation | Initially in a clinic setting and then we get that good feedback and the positive affects, I guess, on the community and we confirm that it's going to be helpful for people |
| Staged implementation | I think, to be honest with you, it need to first roll out in a hospital, okay, and have it as though it's an S8 prescription like anything else. Kind of like even close to how methadone is being done as well so supervised dosing and things like that. |
| **Staged implementation** | Probably in a clinic first and then in a community pharmacy second. I don’t believe it’s a good idea for hospital pharmacy because hospital pharmacy is already restricted in both the number of hours and also how fast they can process patients. Community pharmacy would be the second place, because it is more readily available. But the number one position to supply it would be clinic more so because of specialisation that should be associated with cannabis. So it will require some serious training and also… there might be some criminality initially anyway so probably best at a clinic where there is better security. |
| **Staged implementation** | I think initially clinic. If it ever does get to community pharmacy it would have to be a drug of addition so treated the same or maybe if not more and have a lot more tighter regulations to it the reason for that is we know how much it can be abused. I think initially there would have to be in a clinic first of all to see, something as big as this we would need to understand how the therapeutic effect is, its not as simple as sending someone home and hoping that they get a good therapeutic effect. Something like this where there is a lot that can come from it I think it should be in a clinic to see how it goes. Once or if it does come into community pharmacy it should be a DD, S8 medication if not higher. |
