# Supplementary material for: The Role of Medicinal Cannabis in Clinical Therapy: Pharmacists' Perspectives
Source: PLoS One. 2016 May 12;11(5):e0155113. doi: 10.1371/journal.pone.0155113 (PMC4865212; doi:10.1371/journal.pone.0155113)
Supplement: S2 File — (DOCX) [file pone.0155113.s002.docx]

Internals\\Interviews\\01 (BS) - § 4 references coded [ 7.14% Coverage]

Reference 1 - 0.72% Coverage

Legally, I know that you can actually get it through special access,

Reference 2 - 2.38% Coverage

People who are using it are perhaps not under medical jurisdiction when they use it, because it’s not legal. And probably it would be far more safer and far more effective for it to be made legal rather than the current situation.

Reference 3 - 0.77% Coverage

perhaps it would be more along the lines of a recordable or S8 type of deal.

Reference 4 - 3.27% Coverage

I would imagine that if a scheme for making legal Cannabis available was put through community or through even specialized clinics, it would be along the lines of something like methadone clinics where it’s dispensed, it’s recorded, there’s a special indication and people are being monitored by a specialist

Internals\\Interviews\\02 (BC) - § 2 references coded [ 1.15% Coverage]

Reference 1 - 0.39% Coverage

It’s still illegal in Australia

Reference 2 - 0.76% Coverage

Other countries do have it legal but here, so far, it’s illegal.

Internals\\Interviews\\03 (NI) - § 6 references coded [ 9.55% Coverage]

Reference 1 - 1.71% Coverage

they'll need to be a lot of sort of uhm laws, regulations, and so on and so forth, that need to be met in terms of medicinal cannabis and it's regulation.

Reference 2 - 0.65% Coverage

At the moment I believe in Australia it's still illegal.

Reference 3 - 2.89% Coverage

So just like heroin is illegal, methadone is not an illegal substance when used in the pharmacy and used appropriately and being monitored and the scheduling of it and things like that and I think we should use the same template for medicinal cannabis.

Reference 4 - 1.41% Coverage

It's going to be in a highly regulated medication. It's going to be appropriately. It's going to be monitored and tracked.

Reference 5 - 1.33% Coverage

it's going to be like any other S8 drug and it would be best suited in a community pharmacy setting for access reasons

Reference 6 - 1.56% Coverage

Just like any other S8 it need to be documented in a subsidiary and we work on controlling it. Every mL matters just like we would usually.

Internals\\Interviews\\04 (NE) - § 4 references coded [ 9.70% Coverage]

Reference 1 - 1.47% Coverage

a lot of people have been trying to legalize it uhh but I know they're looking into it but I'm not sure if it has been legalized.

Reference 2 - 2.09% Coverage

it could even be a stage supply type schedule 8 as well. It would sort of, not exactly like our methadone program, but something maybe similar to that which is like a stage supply.

Reference 3 - 2.89% Coverage

I think it would be better if it was done as an authority script because we know it reduces the amount of forgery and things like that because it's hard to forge an authority script. If it doesn't go through we know at least a doctor's been behind it.

Reference 4 - 3.25% Coverage

I think if they start this uhm start it strong and start it, you know, have all the details in place and everything that needs to be done, legalize everything, then there should be no problems but if it's iffy. If there are, could, naw, yeah, maybe it can be, then study it further

Internals\\Interviews\\05 (AH) - § 5 references coded [ 6.05% Coverage]

Reference 1 - 1.16% Coverage

I don't know to much about it's current legal status but I think, as far as I know, that it's still not legal for medicinal or even recreational,

Reference 2 - 1.58% Coverage

I have heard something about it, I'm not sure maybe you can maybe clarify this with me about it's usage in Canberra or something. I heard that people can actually grow it or something in Canberra,

Reference 3 - 0.84% Coverage

they're on a special kind of special program, a special listing just like what we do with S8 medications

Reference 4 - 1.18% Coverage

maybe, if hypothetically in a community pharmacy setting they were to hold it, for them to carry a very limited supply and maybe that might be one.

Reference 5 - 1.29% Coverage

Otherwise you know if there's no support from us as well then there's going to be more barriers in terms of trying to legalize this and to implement this as well.

Internals\\Interviews\\06 (CB) - § 3 references coded [ 6.37% Coverage]

Reference 1 - 0.81% Coverage

I would assume, especially initially it would be S8 and I think that's the appropriate place for it.

Reference 2 - 1.77% Coverage

S8 I think that gives the pharmacist who is giving the medication, some comfort about the level of uhm legislation behind them checking the medication, you know, make sure the doctor's write the prescriptions properly

Reference 3 - 3.79% Coverage

I don't think medicinal cannabis should be like the methadone program. Okay so I think that that's also an area where people are thinking like dose on a daily basis or have that sort of really strict protocols and I'm not sure that's appropriate for that [medicinal cannabis] use because I think that the patients, from our understanding who'll be on this, there's not an abuse issue at all. It's just using it for the medicinal purposes which have been proved beneficial.

Internals\\Interviews\\07 (KT) - § 3 references coded [ 5.05% Coverage]

Reference 1 - 0.72% Coverage

So it's currently illegal but people still do use it for recreational use.

Reference 2 - 2.81% Coverage

there is an avenue for it to be legalized but it's just a matter of being able to supply the cannabis to the correct people and having a way, a system, whereby we filter out the people who might be potentially wanting to abuse it, use it for the wrong purposes and get their hands on some of the stuff.

Reference 3 - 1.52% Coverage

it'd have to be monitored intensively by possibly a pain specialist not just any regular GP or a person that uses it here and there. I has to be properly managed.

Internals\\Interviews\\08 (LM) - § 3 references coded [ 4.67% Coverage]

Reference 1 - 2.07% Coverage

there is so many flaws in the quality and safety of the substance that is taken regarless and thats because tehre are no controls or regulation on it supply. It's making the wrong people rich and is providing an avenue for potentially ruining the lives of young mainly men that take it upon themselves to supply it.

Reference 2 - 0.90% Coverage

like to see pharmacists be heavily involved in the supply of it, for providing the information and also in its regulation of its supply.

Reference 3 - 1.70% Coverage

One of the things that has held pharmacy back in the past would be the absence of a national register or national database, and I guess establishing a nationalised system and accompanying that with the current E-Health Scripts that would help manage this.

Internals\\Interviews\\09 (QTV) - § 2 references coded [ 3.88% Coverage]

Reference 1 - 0.46% Coverage

It's illegal to have marijuana.

Reference 2 - 3.42% Coverage

there's a place for it to become legalized. And so I guess a lot of plants, a lot of medicines have come from plants and so if they can, I don't know, somehow synthesize what's normally in marijuana and use it for medicinal purposes I'm all for it.

Internals\\Interviews\\10 (RM) - § 6 references coded [ 18.54% Coverage]

Reference 1 - 2.15% Coverage

And it's regulated in terms of uhm who's actually able to prescribe it and not any general GP can prescribe it but rather a specialist in a particular area and someone who's done some specific training in that area.

Reference 2 - 1.15% Coverage

All I know is that at the moment it is illegal and they are trying to gain data so that's it's legalized in Australia.

Reference 3 - 3.11% Coverage

Well I think cannabis should be the exact same. If a patient or somebody is found with it without a prescription, without authority, that's when deemed illegal. Whereas if the patient has the authority from a doctor, has a valid reason to be using it, then I feel in that case it's medicinal. It should be legal.

Reference 4 - 3.60% Coverage

as long as pharmacists are aware of the legalities behind it so perhaps there is a specific training for pharmacists to be able to, for example with our QCPP. Say if there's something integrating with that in terms of the pharmacist's procedures and they follow procedures around in cannabis prescriptions and that it is locked in a safe and it's monitored

Reference 5 - 4.76% Coverage

thing I worry about is you put this in a spray, in a liquid form and it's easier to sort of abuse if you think of. Uhm so ideally, for instance with oxycontins you have the new formulation so when a patient try's to sort of use it illicitly, it's very, very hard just like also suboxone, suboxone containing component. So I think that would be a good way to sort of prevent diversion is by putting it into a formulation like that with the science behind it and to make it hard to abuse.

Reference 6 - 3.77% Coverage

if the laws are in place that cannabis can only be prescribed by a specialist and with this particular authority and that authority is, you know, is linked into Medicare so we can prove that is legitimate and we can see the indication that has been written for that patient I think the potential for abuse is a lot less. You know what I mean? It'll just be the way they regulate it.

Internals\\Interviews\\11 (JD) - § 4 references coded [ 5.01% Coverage]

Reference 1 - 1.21% Coverage

if it's going to treat a health problem then it should be legalized but regulated.

Reference 2 - 0.50% Coverage

It depends which state you're in.

Reference 3 - 2.11% Coverage

I think that they should be decriminalized and regulated and then if people have a problem, as in addition, made just like it does with alcohol.

Reference 4 - 1.19% Coverage

I don't see why it would need to be a Schedule 8 medicine. Schedule 4 would be fine.

Internals\\Interviews\\12 (VS) - § 3 references coded [ 4.89% Coverage]

Reference 1 - 2.43% Coverage

I think it's a good option for people that need it. I don't think it should be illegal. I know there's a lot of media about it but I think that it should be available for people that require it pretty much.

Reference 2 - 0.52% Coverage

Probably like an schedule 8 I would think.

Reference 3 - 1.94% Coverage

Or you know like the, I'm not too sure but is it clozapine, like they have like a program where they have to keep in touch with someone, something like that maybe?

Internals\\Interviews\\13 (TH) - § 1 reference coded [ 0.54% Coverage]

Reference 1 - 0.54% Coverage

restrictions behind it in regards to legally obtaining it

Internals\\Interviews\\14 (CS) - § 3 references coded [ 11.84% Coverage]

Reference 1 - 7.83% Coverage

I think it should be legalised medicinally, I don’t think it should be legalised recreationally but I think it needs to be highly regulated for medicinal and there will have to be sort of appropriate auditing processes and things like that and that continue that don’t sort of lapse after the first few years so of the program being implemented so, we know things like the Opioid Substition Program (OSP) that kind of style but then it can’t be too difficult as well because these people are incapacitated to an extent as well, so I think there will definitely be some issues around that to be dealt with.

Reference 2 - 1.02% Coverage

I think it should be a S8 because it will require those increased restrictions

Reference 3 - 2.99% Coverage

should be S8 even incorporating aspects of maybe the clozapine programs and the OSP in terms of recording so whether it’s an online portal like for clozapine but umm regulations associated with dispensing and things like that.

Internals\\Interviews\\15 (TG) - § 3 references coded [ 8.16% Coverage]

Reference 1 - 3.80% Coverage

t’s like the emergency contraceptive pill you know some people are okay some aren’t, in my opinion I would be as long as it’s done the correct way and you know it’s not offered to everyone and you know just like oxycontin which is abused, it would have be to regulated.

Reference 2 - 3.85% Coverage

visible in terms of doctor shopping it would have to be, that will have to come into play and also I think with the digesic regulations where the Doctor needs to provide written authorisation where the pharmacy needs to keep it in a pharmacy for a certain amount of time.

Reference 3 - 0.50% Coverage

I would see it as being a schedule 8.

Internals\\Interviews\\16 (MK) - § 2 references coded [ 3.68% Coverage]

Reference 1 - 2.27% Coverage

I think if the medication is regulated, it is like a proper regulatory framework surrounding it, if it is frequently monitored, all that sort of stuff, I think it would be excellent to be introduced, so yes, so there is.

Reference 2 - 1.41% Coverage

And as long as you know they are always in contact with the Doctor as well as the monitoring all that sort of stuff, I think it would be fine

Internals\\Interviews\\17 (RA) - § 5 references coded [ 9.37% Coverage]

Reference 1 - 1.75% Coverage

it depends on who is going to be able to do it, what the rules are, what’s the cost I suppose, all those sort of things, there is a lot of things for the Government to sort out.

Reference 2 - 1.04% Coverage

I don't think I see it as a S8 based on its use to be honest I see it

Reference 3 - 1.51% Coverage

regulated and make sure, it has to be, an authority prescription they would have to classify it certainly in guidelines and all that sort of stuff

Reference 4 - 2.00% Coverage

A little bit but what can I say, I wouldn’t be all for legalising it at all, I think would be a disaster but medical use for the right purposes I am all for that, I am pretty strong on the way I see that.

Reference 5 - 3.07% Coverage

No I just think it should be done and it’s got to be a national approach obviously the PBS is run nationally so it’s going to have to be a national, there’s no use throughout Australia saying yes and you know whatever, it’s got to be national and something that is agreed on and is done and implemented

Internals\\Interviews\\18 (SK) - § 1 reference coded [ 1.56% Coverage]

Reference 1 - 1.56% Coverage

Yes I believe it should be legalised however there needs to be evidence based guidelines to allow for the prescribing so there needs to be measures in place for it to be available

Internals\\Interviews\\19 (JA) - § 1 reference coded [ 0.51% Coverage]

Reference 1 - 0.51% Coverage

Yes I would be, if it was regulated like Schedule 8.

Internals\\Interviews\\20 (MY) - § 1 reference coded [ 0.51% Coverage]

Reference 1 - 0.51% Coverage

I think it should remain illegal.

Internals\\Interviews\\21 (NL) - § 5 references coded [ 9.16% Coverage]

Reference 1 - 1.98% Coverage

I think private use, it should be decriminalized that’s how I feel about it, because I think it’s a absolute massive waste of resources booking people for smoking a joint, that deserves to be so far away from their criminal justice system. It’s not funny it’s just a huge waste of time,

Reference 2 - 2.03% Coverage

So I can see how with mental health, I can see where decriminalising and has its risks so I think decriminalising it and education campaign, not to dissimilar to what Portugal has done. But stop wasting time on making it as if they are badies they aren't badies they are just less risk averse.

Reference 3 - 1.38% Coverage

so I think it should be decriminalised and that I think it should be really defined indications and it should certainly be script only if it’s going to be a pharmaceutical product available for it.

Reference 4 - 1.14% Coverage

it should be indications and whether that’s on streamline and authority so that the doctor actually has to nominate what indication they are prescribing it for.

Reference 5 - 2.63% Coverage

Like I think once if its established that it’s not abuseable, there is no reason why it shouldn’t just go into an S4 category until its safety is well established and maybe one day if it’s a harmless antinausiant, prochloperazine is now available over the counter and like I would have absolutely no problem recommending a cannabis spray. Once the safety is been established.
