# Supplementary material for: The Role of Medicinal Cannabis in Clinical Therapy: Pharmacists' Perspectives
Source: PLoS One. 2016 May 12;11(5):e0155113. doi: 10.1371/journal.pone.0155113 (PMC4865212; doi:10.1371/journal.pone.0155113)
Supplement: S3 File — (DOCX) [file pone.0155113.s003.docx]

Internals\\Interviews\\03 (NI) - § 1 reference coded [ 4.64% Coverage]

Reference 1 - 4.64% Coverage

It's quite exciting. There's finally going to be a treatment option for those that up until now had no hope and no treatment. And as a pharmacist I guess the patients, you know, health and well being is at the upmost of our priority and for us to be able to help these patients and especially children, to give them a better quality of life and to improve on that is something that basically we work for every day.

Internals\\Interviews\\04 (NE) - § 1 reference coded [ 5.99% Coverage]

Reference 1 - 5.99% Coverage

we're in the field of medicine and helping people and if a certain medicine, no matter what it is, is help the quality of life of a patient therapeutically then there should be no issues or problems with it. Uhm for me, speaking from experience, if a certain medication could have prolonged or made a better quality of life for a cancer patient, you know, that is a big thing a patient that, it can help with their appetites and things like that and that's one of their biggest things that they have to struggle with

Internals\\Interviews\\05 (AH) - § 2 references coded [ 8.76% Coverage]

Reference 1 - 2.92% Coverage

We still have an important role to play. I think ultimately, of course, the prescriber, you know the doctor and the specialists, I think would be playing the major I'd assume. But we still do play an important role as well because especially as advocates to something like this if they were to legalize this I think we do still have like an important role as well.

Reference 2 - 5.84% Coverage

we need to give the community and the government you know the support and uhm the reassurance that we're supportive of this as well. Otherwise you know if there's no support from us as well then there's going to be more barriers in terms of trying to legalize this and to implement this as well. So it is important as well and even from the patient's perspective as well they need to understand that, you know, we're supportive of this and we don't look at this in a negative way, we look at this in a positive light as well, and that they're using this for a legitimate uhm therapeutic purpose then I think definitely our role is quite important as well. Everyone, I suppose, has different roles and we all complement each other.

Internals\\Interviews\\06 (CB) - § 2 references coded [ 4.60% Coverage]

Reference 1 - 1.62% Coverage

we're all pharmacists and so we know the therapeutic benefits of it. You know, we can read the evidence and, you know, we can understand how it works. So I think, yeah we should uhm, we should have a voice.

Reference 2 - 2.98% Coverage

medicinal cannabis should be legalized in Australia and I think that would be great to see Australia being one of the first countries to do that. I know we're not the first but it's certainly being within that the first uhm mover. I think that it will help a lot of people and be beneficial to a lot of people. And I think it's very appropriate to utilize the pharmacy channel.

Internals\\Interviews\\08 (LM) - § 3 references coded [ 6.28% Coverage]

Reference 1 - 0.90% Coverage

like to see pharmacists be heavily involved in the supply of it, for providing the information and also in its regulation of its supply.

Reference 2 - 3.43% Coverage

any pharmacist operates on the guides of evidence based practise and obviously there will be some pharmacist that will hold moral or ethical objection to supplying, that is with anything. But the vast majority of the profession are evidence based professionals. And yeh as pharmacist we are going to have a massive role in supply and education. So we should have a big voice and a say in how its implemented. But in saying that I would want to ensure it is the right people from the industry being involved in that debate.

Reference 3 - 1.94% Coverage

But overall I definitely feel pharmacists are undervalued. Studying for five years and then being payed only $1-$2 more than a hospitality worker is not right for a health care professioanl with so much responsibility and who continues lifelong education and accreditation is just a joke.

Internals\\Interviews\\09 (QTV) - § 2 references coded [ 9.13% Coverage]

Reference 1 - 5.79% Coverage

No not really. I don't think I play a big part in it at the moment although I guess it depends, I guess, depends. Like I've heard stories of it being like a what you would call a marijuana clinics in America and so there's people who have to, in charge or in charge of these clinics and people who have to oversee the use of it and perhaps one day that'll be a part of pharmacy but I don't see that happening any time soon so.

Reference 2 - 3.34% Coverage

I don't quite honestly think that we have to do too much apart from counseling and learning about it and learn about it's properties and side effects and stuff like that. The it's up to other people to determine if it's good for the public.

Internals\\Interviews\\10 (RM) - § 2 references coded [ 7.93% Coverage]

Reference 1 - 3.94% Coverage

cannabis is going to be legalized and it's going to be brought to pharmacy, community pharmacists need to have a say because not all pharmacists are going happy to dispense it and also they should feel involved because really they're going to be providing the access to the patients. I think it's very important that pharmacists actually have their say because they're providing the drug.

Reference 2 - 4.00% Coverage

You're going to have the division, they'll be divided. But as long as uhm a pharmacists does not have to supply it he's able to direct patients where they can get it into pharmacies. they're not completely against it's use and therapy. I think it's the pharmacist duty to care, to direct the patient to a place where they can actually get it because it's not their decision as to what the patient gets.

Internals\\Interviews\\11 (JD) - § 2 references coded [ 4.21% Coverage]

Reference 1 - 2.59% Coverage

do you think it's important that pharmacists speak in this particular debate or do you not see it as their place?

Interviewee: Of course they do if they're going to be involved.

Reference 2 - 1.62% Coverage

We believe that the medicine, the cannabis use needs to be treated as a health problem not a criminal problem.

Internals\\Interviews\\12 (VS) - § 2 references coded [ 9.08% Coverage]

Reference 1 - 4.38% Coverage

I just think it's specialized and it shouldn't, it needs more, it probably needs like, you know, pharmacists and a specialized team rather than, I just think a pharmacist now are already, they have a lot of duties and stress enough. I just think you kind of need, it's sort of like doing methadone. It's kind of like a separate thing so I think it might be too much.

Reference 2 - 4.69% Coverage

of course because we get a lot of first hand info from patients or customers. You know they come and tell you their stories and what's working for them and what isn't working for them. The doctor was too rushed. Didn't have time to listen to me type of thing you know. It seems like their first port of call is the pharmacy now, their local pharmacy where they want a solution and free advice.

Internals\\Interviews\\13 (TH) - § 1 reference coded [ 2.29% Coverage]

Reference 1 - 2.29% Coverage

I wouldn't see why they're against it to be honest with you but I don't know what kind of platform we can but if you can think of one and if we can get the guild or the PSA on top of it maybe of they're all for it the, you know, why not. Don't know what else to say.

Internals\\Interviews\\14 (CS) - § 1 reference coded [ 8.65% Coverage]

Reference 1 - 8.65% Coverage

Yeah I think the pharmacies voice is important because they will be the ones administering it and having to monitor its use and that kind of thing, so I think they should definetly voice that they are for it. But it should be with the appropriate systems and legislation in place and all members of the healthcare team should be involved so it shouldn’t be just you know hospitals or doctors as they are the ones that can do it, it should be you know doctors are trained to prescribe in a certain way then supply it within the community or hospitals and these are the procedures involved in that and aftercare is involved in that as well, so I think it should be continuous.

Internals\\Interviews\\15 (TG) - § 1 reference coded [ 3.56% Coverage]

Reference 1 - 3.56% Coverage

Yes I think it’s important what they should be saying is really up to the individual pharmacist and what they beleive in, but personally I support it and it’s done in the correct way and it’s regulated it’s a Schedule 8 there is no risk, in my opinion.

Internals\\Interviews\\16 (MK) - § 2 references coded [ 7.80% Coverage]

Reference 1 - 7.34% Coverage

Yes if they feel strongly about it and if they are well informed of the trials and the benefits of medicinal cannabis then I think they should because it’s just a step forward and it will help a lot of people and you know a lot of people they try and seek their medication for medicinal purposes but because it’s illegal it makes it very difficult for them and they are up for criminal penalties and things like that so you know sometimes they are there for a general reason and so if you can make that easier for them, if it’s legalised that would help a lot of patients out, yeah I would definitely encourage people to you know step up and support the program, if they can, yeah I think it would be, it’s good progress.

Reference 2 - 0.46% Coverage

and if we have a role in that then that’s huge.

Internals\\Interviews\\17 (RA) - § 5 references coded [ 13.47% Coverage]

Reference 1 - 3.44% Coverage

it will be interesting, but I can’t think of a better place to do it, any pharmacy really should be able to do it, should probably have some pharmacists that might not want to do it but yeah I don’t know, it depends how they are going to regulate it, it depends what the rules are going to be and so as it is going to happen, and how it happens.

Reference 2 - 2.55% Coverage

Seriously I am more than happy to dispense it, well as long as it’s really clear, the rules and regulations, as long as we think we are doing some good, I mean what’s the Doctor’s moto I think that’s what our moto basically is as well, first do no harm.

Reference 3 - 1.25% Coverage

If we think they are going to benefit and they can’t find relief from anything else, then I am all for it, that’s my opinion

Reference 4 - 4.72% Coverage

Well I think it’s very important if they are going to part of the delivery process, yeah definitely, if we are not then it probably doesn’t matter but I would have thought the only way of making it happen, you wouldn’t want to have shops like they do in Colorado you know just giving it out willy nilly, I think Australia is not looking at doing that, for the right reason, I think the obvious thing is that pharmacy is the place to deliver it I would have thought.

Reference 5 - 1.50% Coverage

Who else can do it, no one else can do it, Doctor’s I don’t think would want to do it, I just think no one else can do it really other than pharmacists.

Internals\\Interviews\\18 (SK) - § 2 references coded [ 8.17% Coverage]

Reference 1 - 3.44% Coverage

cause it’s going to involve pharmacy whether we like it or not, because you know we don’t know how it is going to be supplied but let’s say it is supplied through community or hospital you know it’s going to involve a pharmacist and you know because it will involve us, we need to have our input into that matter I think that is very important you know we are most likely the ones dispensing supply s

Reference 2 - 4.73% Coverage

there are a lot of other issues to think about with this and implications which are not necessarily medical in nature, that do affect pharmacists so you know a lot of things, so we need to have our input, there needs to be a forum where all key stakeholders who are involved in this issue need to have a discussion and they need to raise issues that are going to affect them or their profession and consideration needs to be made so I think that’s very key, I think that is something that should not be rushed into, so that would be my thought on that.

Internals\\Interviews\\19 (JA) - § 1 reference coded [ 2.43% Coverage]

Reference 1 - 2.43% Coverage

I would say it’s not important at this point in time, because as far as I am aware there isn’t enough evidence based information readily available for us to make an educated decision, as far as I am aware it may be out there but I am not aware of it.

Internals\\Interviews\\20 (MY) - § 1 reference coded [ 3.44% Coverage]

Reference 1 - 3.44% Coverage

Yes definitely, pharmacists do have a key role I guess that’s the same as you know involved in the medication as well, so I think definitely there is an important role in e

Internals\\Interviews\\21 (NL) - § 3 references coded [ 9.03% Coverage]

Reference 1 - 2.73% Coverage

I think the future pharmacy I hope the future for pharmacy is more professional interaction, I hope that’s just the way – and I know with the forward dispensing operation that we’re going here, having the pharmacist in the shop means we can they write the forefront 24 hours a day. None of them are hiding into the, therefore that interaction to help people at here and manage health services

Reference 2 - 2.06% Coverage

I rate pharmacists so like in particularly the young pharmacists coming through I’d 22 interns in the last 20 years and they have been been fantastic. So I think interns are more capable and they’ve ever been now. And I think I was handing them responsibilities these are really good thing to do,

Reference 3 - 4.24% Coverage

nd so I reckon, I think they are really capable I said rather, having said that experience does count, it absolutely counts. But there is no reason why they can’t, you can’t have a really productive pharmacy practice course which covers up, which makes every graduate perfectly capable of managing something like this. And I don’t think there is too many for them not to be capable of doing all of them and if that’s doing medscheck or handling medicinal cannabis or smoking sensation program like. I'm unafraid of giving pharmacist responsible because I think they are better than they have ever been now.
