# Supplementary material for: The Role of Medicinal Cannabis in Clinical Therapy: Pharmacists' Perspectives
Source: PLoS One. 2016 May 12;11(5):e0155113. doi: 10.1371/journal.pone.0155113 (PMC4865212; doi:10.1371/journal.pone.0155113)
Supplement: S4 File — (DOCX) [file pone.0155113.s004.docx]

Internals\\Interviews\\01 (BS) - § 3 references coded [ 3.66% Coverage]

Reference 1 - 0.55% Coverage

see if we can have some kind of standard formulations

Reference 2 - 1.80% Coverage

committee should recommend a standard formulation containing those moiety be prepared and then that’s the one standard formulation that’s made available nationally.

Reference 3 - 1.31% Coverage

I would strongly recommend that there be standardised formulation that are actually trialled or tested or made available.

Internals\\Interviews\\03 (NI) - § 1 reference coded [ 1.48% Coverage]

Reference 1 - 1.48% Coverage

Medicinally cannabis will become purified in it's form and will only have the component required to treat certain conditions.

Internals\\Interviews\\04 (NE) - § 1 reference coded [ 1.54% Coverage]

Reference 1 - 1.54% Coverage

So if it's formulated in a way that a community pharmacy can handle it and can deal with it then yeah why not be in a community setting

Internals\\Interviews\\05 (AH) - § 1 reference coded [ 2.56% Coverage]

Reference 1 - 2.56% Coverage

this stage we might as well use uhm evidence based medicine and what they currently uhm have standardized, I suppose, formulations. When we know more about it because all of these things as well we see you know the development of all these physical formulations in the current different way. So why not if they can be?

Internals\\Interviews\\06 (CB) - § 3 references coded [ 4.87% Coverage]

Reference 1 - 0.94% Coverage

to insure that there's an understanding of, you know, the dosages and the quantities rather than, you know, a guess.

Reference 2 - 1.52% Coverage

it ensures that patients are getting the therapeutic dose that they require and then not playing doctors or pharmacists themselves, but they actually have a health care team behind them.

Reference 3 - 2.41% Coverage

inherently, you know, I guess there's a reason why people smoke cannabis. And perhaps that's the way that it's more effective. I'm not sure. It would be interesting to know the pharmacology behind it and see which one's better. Perhaps, you know, there's ways it could be a vapor that people inhale.

Internals\\Interviews\\07 (KT) - § 1 reference coded [ 1.50% Coverage]

Reference 1 - 1.50% Coverage

So they're all smoking. So obviously that might not be the dosage form that they're going to choose. Simply being maybe a tablet for a different sort of form.

Internals\\Interviews\\09 (QTV) - § 4 references coded [ 8.04% Coverage]

Reference 1 - 1.63% Coverage

I guess it depends on how they us the, how they, I guess, extract it and use what sort of form that eventually have it in

Reference 2 - 2.91% Coverage

It's just like a tablet or a spray that you use like there's so many different forms of medicines and different sort of dosage forms so it's just like that. It's seen like a puff or a spray. Something like that.

Reference 3 - 1.69% Coverage

Yeah, I guess tablets would be the best if they can make it into a tablet and so it's just like dispensing a box of tablets.

Reference 4 - 1.80% Coverage

Just seeing that there is evidence for it so if they can somehow make it to a dosage form that's beneficial then ahm I'm all for it.

Internals\\Interviews\\11 (JD) - § 2 references coded [ 5.14% Coverage]

Reference 1 - 3.27% Coverage

I think in the compounding pharmacy setting it would tend to be the actual alkaline. You wouldn't be giving the plant because you need to have a regulated, the whole idea of regulation is that you had to get the correct dose.

Reference 2 - 1.87% Coverage

As long as the active ingredients are produced or packaged by a TGA licensed facility then I don't see why there'd be a problem.

Internals\\Interviews\\12 (VS) - § 1 reference coded [ 1.07% Coverage]

Reference 1 - 1.07% Coverage

If it were in a capsule form I guess that would be, feel more confortable working with it.

Internals\\Interviews\\13 (TH) - § 1 reference coded [ 3.02% Coverage]

Reference 1 - 3.02% Coverage

even if they changed the formulation I don't think that it would, I guess, improve these problems that we would encounter. The only thing I can think of is they could change it like the oxycontin new formulation in which case if you try to crush it, whatever, you can't physically do it in which case they wouldn't be able to abuse it.

Internals\\Interviews\\14 (CS) - § 1 reference coded [ 3.25% Coverage]

Reference 1 - 3.25% Coverage

I don't think I would mind any form I think with like a set dose or a set sort of measurement that you can't adjust, the difficulty would be where it is prescribed in different quantities... so as long as it’s a flexible sort of form I don’t mind what it is.

Internals\\Interviews\\15 (TG) - § 1 reference coded [ 2.57% Coverage]

Reference 1 - 2.57% Coverage

you want it to absorb quite fast so in a tablet capsule form it may not be absorbed faster, I think a spray under the tongue is quite a fast option or maybe even a patch, I don’t know...

Internals\\Interviews\\16 (MK) - § 2 references coded [ 6.86% Coverage]

Reference 1 - 4.25% Coverage

Yeah it is a bit tricky, I don’t think it matters too much, it would be good if both are available, you do have your pros and cons with both so in terms of like the spray its absorbed a bit better better but it may not get the right dosage time, capsules are a bit slower so things like that, I think there are different factors that you have to take into account but I think both forms if they are available would be good.

Reference 2 - 2.61% Coverage

Especially when you get it from the black market and things like that, you might not even get pure substances, so getting synthetic forms of it can be way more dangerous than the natural ones, so I think getting the right medication, the right form of it

Internals\\Interviews\\17 (RA) - § 1 reference coded [ 2.63% Coverage]

Reference 1 - 2.63% Coverage

if you look the DD Cabinet now you have got all sorts of forms you have got patches, you have got tablets you have got suppositories, you have got you know, we have got sprays on the shelves, whatever form works the best I suppose, I don’t see that as an issue.

Internals\\Interviews\\18 (SK) - § 1 reference coded [ 3.77% Coverage]

Reference 1 - 3.77% Coverage

So liquid form, from my point of view like that would be more versatile in different demographics, different age groups, so it’s more, obviously as I said it has to be evidenced and I can’t really I can’t really comment on what’s appropriate and what’s not because I am not in a position to, but what I can say is that it needs to be that evidenced in order to which form of administration would provide the best outcome for the patient,

Internals\\Interviews\\19 (JA) - § 1 reference coded [ 2.35% Coverage]

Reference 1 - 2.35% Coverage

Look I think any formulation will be fine, as far as dosage goes I have no idea what would be an appropriate dosage or not, dosage forms though I suppose anything other than smoking would make it seem more medicinal less recreational.

Internals\\Interviews\\21 (NL) - § 1 reference coded [ 0.30% Coverage]

Reference 1 - 0.30% Coverage

so I not afraid of different formulations.
