# Supplementary material for: The Role of Medicinal Cannabis in Clinical Therapy: Pharmacists' Perspectives
Source: PLoS One. 2016 May 12;11(5):e0155113. doi: 10.1371/journal.pone.0155113 (PMC4865212; doi:10.1371/journal.pone.0155113)
Supplement: S5 File — (DOCX) [file pone.0155113.s005.docx]

Internals\\Interviews\\01 (BS) - § 4 references coded [ 2.59% Coverage]

Reference 1 - 0.73% Coverage

always imagining a place like Nimbin or you know a hippie crowd using it

Reference 2 - 0.89% Coverage

I do think that in the general public, there is definitely stigma associated with it.

Reference 3 - 0.67% Coverage

there is a level of stigma, media wise and public labelling wise

Reference 4 - 0.30% Coverage

there’s the issue of stigma,

Internals\\Interviews\\02 (BC) - § 1 reference coded [ 1.92% Coverage]

Reference 1 - 1.92% Coverage

The overcasting or the shadowing of its potential for good use by its recreational use and the bad side effects of that is what's affected progress in this area.

Internals\\Interviews\\03 (NI) - § 2 references coded [ 7.35% Coverage]

Reference 1 - 2.68% Coverage

the name would not be cannabis so maybe would loose some of the stigma that's attached to it if we loose that name and give it a medicinal term and educate people that it isn't in fact cannabis in the way that we know it. It's been purified.

Reference 2 - 4.67% Coverage

lack of education mainly and without educating the patient as to when we say we want to make cannabis legal if obviously it's on the news, "Do you want to make cannabis legal or not?" It's a bit like, "Ahh yes... I don't get it... everyone can smoke marijuana and grow it in the back yard." That's the first thought that comes to a general person's mind who's not a pharmacist or maybe even if they're a pharmacist.

Internals\\Interviews\\04 (NE) - § 1 reference coded [ 3.50% Coverage]

Reference 1 - 3.50% Coverage

if people don't understand that maybe they should go and see a part of the care, a hospital, and see what these people are going through and then make that decision if they are quick to judge. If that's the case then further education, even for our pharmacists or our health professionals is needed.

Internals\\Interviews\\05 (AH) - § 1 reference coded [ 1.95% Coverage]

Reference 1 - 1.95% Coverage

That stigma is pretty much dying now in terms of it's usage. I'm not saying there isn't, there is definitely a stigma but it's stigma is becoming less and less prominent in todays society because of this potential you know positive affects.

Internals\\Interviews\\06 (CB) - § 2 references coded [ 3.76% Coverage]

Reference 1 - 0.71% Coverage

it becoming legalized is really, really important. One it takes the stigma away from it

Reference 2 - 3.06% Coverage

I think there's always going to be a stigma attached with that. I think as soon it becomes uhm you can use it for therapeutic treatment and the evidence behind that and I'm sure there'll be a public awareness campaign behind that. I think the stigma will be reduced, you know, substantially. I think cannabis is milder drug than the other illicit drugs that are on the market as well.

Internals\\Interviews\\07 (KT) - § 3 references coded [ 8.13% Coverage]

Reference 1 - 2.26% Coverage

kept in a clinic specific for just cannabis or marijuana, probably in a hospital and if there are areas which a lot of people are relying on the medicine for it's medicinal uses then possibly just a clinic just devoted to marijuana, yeah.

Reference 2 - 2.39% Coverage

f it does become legalized and we do use it for it's medicinal properties the stigma around it would kind of be uplifted, it would be a lot lighter. I guess when you say marijuana people just think of you know people holding a little stick and smoking it.

Reference 3 - 3.48% Coverage

it's heard on the news and it's usually involving he police and, you know, big heists and all sorts of criminal offenses regarding the substance itself. That's the reason why we have that sort of stigma cause all you hear about it is the bad stuff because it's not legal. I guess it's more connected to criminal activity and abuse rather than medicinal things here.

Internals\\Interviews\\08 (LM) - § 4 references coded [ 5.61% Coverage]

Reference 1 - 1.80% Coverage

And if someone does have something against mediicnal marijuana it would stem more so from an upbringing and cultural thing ...its a massive generalisation i know but it is seen in a wide range of issues and people don’t want to accept something new, different or a change.

Reference 2 - 1.32% Coverage

young people that you meet are already used cannabis, and they're quite open minded. So that’s the younger people but there is also like a lot of resistance and hesitance from certain cultures like,

Reference 3 - 1.44% Coverage

she was for a better word fearful due to the heavy stigma attached to cannabis. In some ways quite rightly so the mindset that by the way it’s been approached and has been abused and something that people get fucked up on

Reference 4 - 1.06% Coverage

Its just the more normalised it is for cannabis in regards to its moderate beneficial use be it medicianlly or recreationally the less of a stigma it will carry.

Internals\\Interviews\\09 (QTV) - § 1 reference coded [ 5.82% Coverage]

Reference 1 - 5.82% Coverage

Yeah, I guess having it like prescribed or something like that from doctors and having it administered in the pharmacy that would definitely decrease the stigma. I guess that adds a bit of creditability to it instead of having like in the US where they have marijuana clinics where it's just people who come in, they buy their pot or whatever. If it comes in like a pharmacy then it might change people's views of it.

Internals\\Interviews\\10 (RM) - § 2 references coded [ 6.68% Coverage]

Reference 1 - 4.54% Coverage

to be honest I haven't heard too many people uhm express negative view about it. We've actually had some patients ask about do you know where it's at this stage because you know they might have a child. There is one child we know, we won't name the name or anything, but they have some kind of neurological condition and the professor has actually mentioned something about cannabis and uhm hopefully that will be a future treatment for their condition.

Reference 2 - 2.14% Coverage

I don't think there's that much negative stigma. You know, even if there is uhm people will get over it. You know what I mean? Once they see it's actually benefits in their community it might pay their perspective.

Internals\\Interviews\\11 (JD) - § 3 references coded [ 6.66% Coverage]

Reference 1 - 2.36% Coverage

Well you wouldn't call it cannabis for a start so one would know what it is so I don't know. There are a lot of people that think it should be decriminalized anyway.

Reference 2 - 1.44% Coverage

I would say people mainly are against it because it is illegal and they don't want to break the law.

Reference 3 - 2.86% Coverage

that we pharmacists need to be a lot more up front about the health issues involved with any sort of drug addiction and that we need to be the ones that then refer people on and not be judging people.

Internals\\Interviews\\12 (VS) - § 2 references coded [ 6.59% Coverage]

Reference 1 - 4.12% Coverage

I don't feel there is alot of stigma to be honest. I think there's more like a push for it to be available. I've seen a lot of stories where it's been beneficial. So maybe with my health background it's like I don't see it that way. Like I see it as helpful but maybe just who's without a health background they might have a different view I guess.

Reference 2 - 2.47% Coverage

if the general public have a negative view on it it would be more because of lack of understanding or experience or that don't know anyone that's been in that situation or even had like terminal illnesses

Internals\\Interviews\\14 (CS) - § 2 references coded [ 5.09% Coverage]

Reference 1 - 3.63% Coverage

if you do it in community settings you will take away a lot of the stigma associated with it as well, because we have seen with happens with just umm the OSP in clinics and hospitals they are not best environment to go to and that’s the same thing for a person who palliative or in pain.

Reference 2 - 1.46% Coverage

I think it’s slowly, slowly going away as a stigma I think it’s becoming more accepted as part of normal culture,

Internals\\Interviews\\15 (TG) - § 2 references coded [ 7.76% Coverage]

Reference 1 - 1.70% Coverage

I think people perceive it to be for recreational use, people who are unemployed and use it for their own personal gain

Reference 2 - 6.06% Coverage

The media really you know play it out to be soley an illicit drug and you see all those stories of plants being grown and discovered in rural areas and use for personal gain and sold on the black market and even areas like Nimbus which its almost legalised and so there’s pockets around Australia where it’s almost ignored by the Police and authorities but that’s again that might not be the case, but that’s how I perceive it.

Internals\\Interviews\\16 (MK) - § 2 references coded [ 4.50% Coverage]

Reference 1 - 1.94% Coverage

I think it does have a lot of negative connotations in the community, so often when you say the word cannabis people often just think about you know the negative aspects and side effects.

Reference 2 - 2.56% Coverage

not educated enough about it so they may still have that stigma about it because time goes on and as you know progress occurs with it, I think they will be more informed and I think the stigma will come down, but I do think it’s definitely still there.

Internals\\Interviews\\18 (SK) - § 2 references coded [ 7.64% Coverage]

Reference 1 - 4.58% Coverage

w there is stigma with it, so anyone you know I know that anyone uses drugs especially illicit drugs you know society looks down on them it’s just the way it is, not everyone is like that, I can’t generalise it, just from my observations because obviously dealing with these methadone patients, so I know that a lot of people look down on them and treat them badly, we don’t really know their situation and a lot of people jump to and they don’t know how these people got into these situations in the first place, so there is a stig

Reference 2 - 3.06% Coverage

h I think our generation, younger generation are becoming more accepting and are not so black and white, we see the grey areas more and yes these things can have potential in a sort of medical treatment, rather than just recreational use, but I think the older generation may view it differently, so yeah that would be my view on it, yes there is stigm

Internals\\Interviews\\19 (JA) - § 1 reference coded [ 0.95% Coverage]

Reference 1 - 0.95% Coverage

Oh I think it is the fact that it’s illegal I mean the stigma will stay whilst ever it is illegal.

Internals\\Interviews\\20 (MY) - § 1 reference coded [ 1.87% Coverage]

Reference 1 - 1.87% Coverage

: I think so yeah like a stigma not including the medical use, people will be thinking that it is mainly for recreational u

Internals\\Interviews\\21 (NL) - § 2 references coded [ 1.66% Coverage]

Reference 1 - 0.44% Coverage

I absolutely believe that there is stigma associated with it.

Reference 2 - 1.22% Coverage

I think pharmacist see cannabis as being a Central Nervous Depression with a psychoactive component, and whereas consumers I think just see it as something to get high with.
